# SMURF-seq: efficient copy number profiling on long-read sequencers

**DOI:** 10.1186/s13059-019-1732-1

**Published:** 2019-07-08

**Authors:** Rishvanth K. Prabakar, Liya Xu, James Hicks, Andrew D. Smith

**Affiliations:** 1 Quantitative and Computational Biology Section, Department of Biological Sciences, University of Southern California, 1050 Childs Way, Los Angeles, 90089 USA; 2 Michelson Center for Convergent Bioscience, University of Southern California, 1002 Childs Way, Los Angeles, 90089 USA

**Keywords:** Long-read sequencing, Nanopore sequencing, Copy number variation, Read-counting applications

## Abstract

We present SMURF-seq, a protocol to efficiently sequence short DNA molecules on a long-read sequencer by randomly ligating them to form long molecules. Applying SMURF-seq using the Oxford Nanopore MinION yields up to 30 fragments per read, providing an average of 6.2 and up to 7.5 million mappable fragments per run, increasing information throughput for read-counting applications. We apply SMURF-seq on the MinION to generate copy number profiles. A comparison with profiles from Illumina sequencing reveals that SMURF-seq attains similar accuracy. More broadly, SMURF-seq expands the utility of long-read sequencers for read-counting applications.

**Electronic supplementary material:**

The online version of this article (10.1186/s13059-019-1732-1) contains supplementary material, which is available to authorized users.

## Background

In the last decade, massively parallel high-throughput short-read sequencing has revolutionized the efficiency and breadth of applications for DNA sequencing [1]. These high-throughput sequencing methods produce millions to billions of short reads in a single run and have led to the development of many applications that depend on “read-counting” to measure the abundance of specific sequences in a sample. Examples include RNA-seq, ChIP-seq, and whole genome copy number profiling. Recently, long-read technologies have been developed that are filling the gap left by short-read sequencers in applications such as genome assembly [2, 3], which benefit from connecting more distant sequences within a contiguous molecule. Among these, the MinION instrument, from Oxford Nanopore Technologies, is highly portable and inexpensive and has shown its unique value for analysis outside of central sequencing facilities [4]. Long-read sequencers such as the MinION typically produce vastly fewer reads from a sequencing run and are therefore less efficient in applications that use sequenced reads purely as a means to count molecules. However, these technologies have the enormous advantage of operating in near real-time, with a turnaround time that can be measured in hours for some applications, rather than days or weeks.

Copy number variation (CNV) has been used successfully to understand a variety of diseases [5]—notably cancers, which exhibit both extreme variation and recurrent trends that can be used for diagnostics and personalized approaches to treatment. For example, the amplification and loss of certain genes, such as *RB1* deletion and *MYCN* amplification in retinoblastoma, can be prognostic or even predictive for treatment [6]. High-throughput short-read sequencing has been extremely effective in copy number profiling of cancers [7], including profiling single tumor cells [8]. However, for many potential users, the efficiency of high-throughput short-read sequencing in CNV analysis is determined by the availability of instruments and need for heavy multiplexing to hit reasonable cost per profile. A sequencing core is typically involved and an individual profile must wait for a “full” run before it can be processed. The MinION sequencer has an accessible buy-in and is easy to use. Unfortunately, the MinION has optimal nucleotide throughput when producing reads that are orders of magnitude longer than needed for CNV profiling.

To make full use of the advantages offered by the MinION sequencer, we introduce sampling molecules using re-ligated fragments (SMURF)-seq, a protocol to efficiently sequence short DNA molecules on a long-read sequencer. The strategy of SMURF-seq is to concatenate short fragments into very long molecules (∼8 kb) prior to sequencing. The concept of ligating short molecules together prior to sequencing was introduced in serial analysis of gene expression (SAGE) [9] and then subsequently used in short multiply aggregated sequence homologies (SMASH) for CNV profiling using Illumina short-read technology [10] and ConcatSeq for target enrichment workflows on PacBio machines [11]. SMURF-seq differs from these methods in that the fragmented and re-ligated molecules are substantially longer, it allows for variable fragment lengths as permitted by long-read sequencing, and the number of fragments within each read is substantially greater. Here we describe the details of the SMURF-seq approach and demonstrate the accuracy of this approach for CNV profiling.

## Results

### The SMURF-seq approach to sequence short molecules

**Fig. 1:**
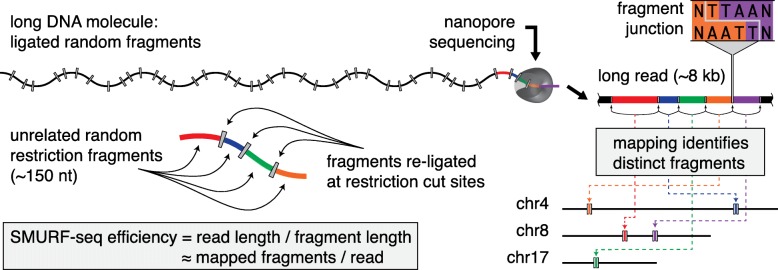
SMURF-seq efficiently sequences short fragments of DNA for read-counting applications with a reference genome on long-read sequencers and yields up to 30 countable fragments per sequenced read. SMURF-seq sequences short DNA molecules by generating long concatenated molecules from these. SMURF-seq reads are aligned by splitting them into multiple fragments, each aligning to a distinct region in the genome

The SMURF-seq protocol involves cleaving the genomic DNA into short fragments. These fragmented molecules are then randomly ligated back together to form artificial long DNA molecules. The long re-ligated molecules are sequenced following the standard MinION library preparation protocol. After (or possibly concurrent with) sequencing, the SMURF-seq reads are mapped to the reference genome in a way that simultaneously splits them into their constituent fragments, each aligning to a distinct location in the genome (Fig. 1).

More specifically, genomic DNA is fragmented using restriction enzymes that result in short fragments, with length just sufficient for an acceptable rate of uniquely mapping fragments in the reference genome. For the human reference, 100 bp is a reasonable length. In our applications, we tested SaqAI and Hin1ll restriction enzymes, which produce molecules with mean lengths of 150.2 bp and 208.9 bp, respectively. The fragmented DNA molecules are then ligated randomly to form longer molecules using T4 DNA ligase enzyme (Additional file 1: Figure S1). The resulting long DNA molecules are sequenced following standard MinION library preparation protocols (in our experiments we used two different protocols). The SMURF-seq protocol is completely enzymatic and takes less than 90 min to complete (Additional file 1: Figure S2 and Additional text 1.1). We also tested dsDNA Fragmentase enzymes (New England Biolabs) and acoustic shearing (Covaris) to fragment DNA. However, these methods require an additional end-repair step after fragmentation and the ligated molecules failed to reach the lengths we obtained by using restriction fragmentation (Additional file 1: Additional text 1.2).

The reads sequenced using SMURF-seq can be mapped to a reference genome by first identifying short matches within the reads, corresponding to parts of the individual fragments, and then extending those to locate fragment boundaries. This is handled nicely using the seed-and-extend paradigm implemented in many existing long-read mapping tools. Although none of these tools were designed to align SMURF-seq reads, several long-read aligners such as BWA-MEM [12], Minimap2 [13], and LAST [14] include steps designed for split-read alignment, which can be leveraged for aligning SMURF-seq reads. We evaluated these tools on simulated SMURF-seq data generated by concatenating random fragments from real Oxford Nanopore reads. This emulates idealized SMURF-seq reads. Within the simulated reads, the boundaries of each fragment are known a priori, as are their mapping locations when in the context of their original long reads. We used this information to evaluate mapping tools in terms of (1) how well they identify fragments purely for the purpose of counting molecules, which is the primary information used in CNV analysis, and (2) how well they identify individual mapping bases within reads. After mapping these reads, we calculated precision and recall for identifying both the correct fragment locations, and the individual mapping bases within the fragments (i.e., the correct fragment boundaries). Using this simulation setup, we determined the optimal Smith-Waterman alignment score for use with SMURF-seq reads (Additional file 1: Additional text 2). Based on these results, BWA-MEM outperformed other tools, and thus, we used BWA-MEM to align SMURF-seq reads (Additional file 2: Additional table 1 and 2). Briefly, BWA-MEM uses short seed hits originating from different parts of the long reads (and therefore, in our application, different fragments within those long reads), to form clusters of seed hits in the reference genome. Nearby clusters are joined, and then extended, eventually resulting in (for most fragments) one alignment per fragment. In our analysis, we employed BWA-MEM without any modifications to optimize identification of fragment boundaries. According to our simulations, this mode of operation may not perfectly identify fragment boundaries, but performs well when identifying mapping locations of the individual fragments, which is the information passed to subsequent steps in our analysis.

### Generating higher fragment counts in a sequencing run

CNV profiling, and read-counting in general, can be done on nanopore sequencers with long reads following the standard sequencing procedure [15]. A typical Oxford MinION sequencing run generates approximately 500k reads (length ∼8 kb) [2, 16]. Read-counting applications in general do not benefit from longer reads beyond what is necessary for unique mapping to the reference genome. In these applications, for any fixed number of nucleotides sequenced, more information is obtained if those nucleotides are organized as more DNA molecules, rather than longer contiguous fragments.

In general, for a given sample of DNA, a nanopore instrument will generate more reads if the corresponding molecules are shorter. Once a molecule is loaded into a pore, the time spent sequencing is less for shorter reads. In addition, for a fixed amount of DNA, shorter molecules result in higher molar concentration when loaded onto the machine, increasing the rate at which each pore captures molecules [17, 18]. We verified this rationale by sequencing short DNA molecules (restriction enzyme digested normal diploid genome) using the Oxford MinION instrument. The sequencing run produced 2.58 million reads with a mean read length of 630.93 bp (Additional file 1: Figure S3 and Additional text 3.1). Using the same instrument, the SMURF-seq runs, we report here average 6.2 million mapped fragments per run, which is substantially more fragments than directly sequencing short reads (Additional file 1: Additional table 3).

### Accurate CNV profiles using SMURF-seq

To demonstrate the utility of SMURF-seq, we generated CNV profiles of normal diploid and highly rearranged cancer genomes. The mapped fragments were grouped into variable length “bins” across the genome and bin counts were used to generate CNV profiles as described in [19, 20].

**Fig. 2:**
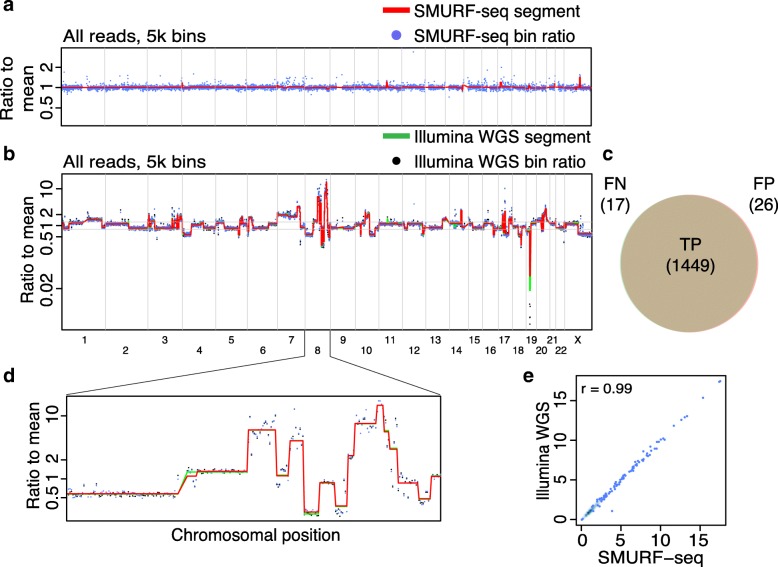
Accurate copy number profiles with SMURF-seq. **a** CNV profile of a normal diploid genome. Each blue point is a bin ratio to mean and the red line is the segmented bin ratio. **b** Superimposed CNV profiles of SK-BR-3 genome generated using SMURF-seq and Illumina WGS reads. **c** Venn diagram illustrating the accuracy of event calls using SMURF-seq compared with Illumina WGS. **d** Zoom-in of copy number changes on chromosome 8. **e** Scatter plot of bin ratio of SK-BR-3 genome using SMURF-seq and Illumina WGS reads. Pearson correlation of the data is shown

We sequenced a normal diploid female genome with SMURF-seq, resulting in 270.8k reads (mean read length of 6.75 kb) in a single run. These reads were split into 7.28 million fragments (26.87 mean fragments per read). A CNV profile for this normal diploid genome, with the expected (approximately flat) appearance can be seen in Fig. 2a (and Additional file 1: Figure S4). We verified that the SMURF-seq procedure behaves similarly using the Rapid Sequencing Kit (Additional file 1: Figure S5). Next, we applied SMURF-seq to the breast cancer line SK-BR-3, generating 147.0k reads with mean length of 7.62 kb, which were split into 4.52 million fragments (30.78 mean fragments per read). We then obtained a CNV profile using 5000 bins, corresponding to an average bin size of approximately 600 kb (Fig. 2b; Additional file 1: Figure S6).

To provide a quantification of accuracy in terms of individual CNV events, we conducted whole-genome sequencing (WGS) on the same SK-BR-3 using Illumina (5.56 million reads; 130 bp, single-end). We used this to define a ground truth by calling CNV events for each of the pre-defined bins (both amplifications and deletions) based on segmented signal with a cutoff of 1.25/0.8 (Fig. 2b) [6, 21]. This resulted in 1466 events (886 amplifications, 580 deletions) from 4953 bins. We then called events using the identical procedure with SMURF-seq data from the same SK-BR-3 sample. The precision and recall for SMURF-seq relative to the Illumina calls was 0.982 and 0.988, respectively (Fig. 2c). Figure 2d shows a zoom-in of a region with extreme copy number alterations. The bin ratios for the Illumina WGS and the SMURF-seq profiles are highly correlated (Pearson *r* = 0.99; Fig. 2e). Replicates for these genomes show a high degree of reproducibility for these profiles (Additional file 1: Figure S7 and S8).

We also generated higher-resolution CNV profiles at 20,000 and 50,000 bins, corresponding to an average of approximately 150 kb and 60 kb in length respectively (Additional file 1: Figure S9a, b). The profiles obtained at these resolutions have a high correlation with the profiles obtained using Illumina WGS (Pearson *r*> 0.97; Additional file 1: Figure S9c, d). Using SMURF-seq also generates fragments at a faster rate than sequencing short molecules directly (Additional file 1: Figure S10), and the CNV profile with reads generated in the first 45, 90, and 180 min of sequencing had a high correlation to the profile with reads from the complete run (Pearson *r*> 0.98; Additional file 1: Figure S11).

### Concordant profiles from fewer countable fragments

**Fig. 3:**
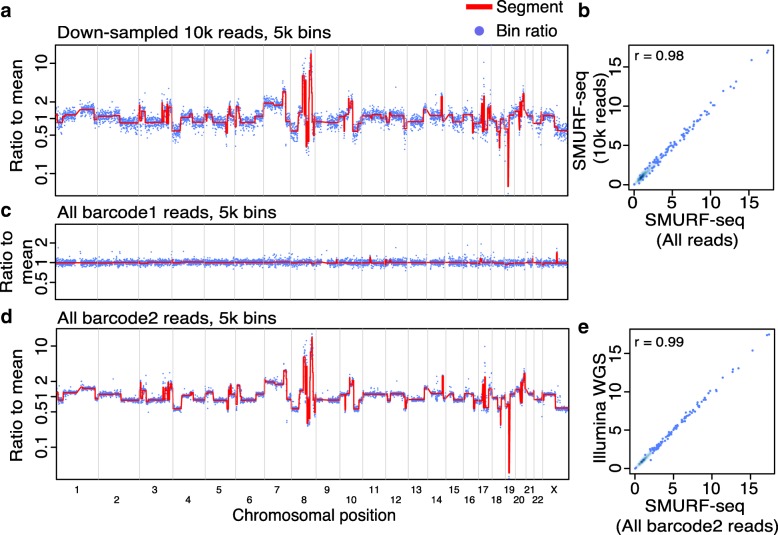
Multiple SMURF-seq CNV profiles by multiplexing in a single run. **a** CNV profile of SK-BR-3 genome with down-sampled 10k SMURF-seq reads. **b** Scatter plot of normalized bin counts of the original SMURF-seq data and data down-sampled to 10k SMURF-seq reads. Pearson correlation of the data is shown. **c** CNV profile of barcode01 (Normal diploid genome) reads. **d** CNV profile of barcode02 (SK-BR-3 cancer genome) reads. **e** Scatter plot of bin ratios of SK-BR-3 genome using multiplexed SMURF-seq and Illumina WGS reads

Several cancer-related studies have employed CNV profiling based on low-coverage WGS [22, 23]. It has previously been demonstrated that 250k reads are sufficient for accurate genome-wide CNV profiling of single cells [24]. At the same time, the CNV profiles from a population of cells has been shown to have a high correlation with single-cell profiles [8, 24]. We reasoned that using 250k fragments for CNV profiling using a population of cells would give useful profiles if they remained sufficiently accurate. By down-sampling our SMURF-seq data, we verified that 10k reads, approximately 250k fragments, result in highly correlated CNV profiles (Pearson *r* = 0.98; Fig. 3a, b).

Given the total capacity of the MinION instrument, this indicates that multiple samples can effectively be barcoded and multiplexed in a single sequencing run. To verify this, we sequenced two DNA samples (normal diploid female and SK-BR-3) in a single run (Additional file 1: Figure S12). These samples were processed with SMURF-seq protocol and then barcoded following the standard library construction. After demultiplexing and mapping the reads, the diploid genome had a CNV profile as expected (Fig. 3c) and the SK-BR-3 CNV profile was nearly identical to the profile obtained using Illumina WGS (Pearson *r* = 0.99; Fig. 3d, e).

## Discussion and conclusion

Our results demonstrate that SMURF-seq can generate more information for CNV analysis in a single run of the Oxford MinION sequencer, compared with either producing long reads in the usual way or direct short-read sequencing on the same instrument. This increased information is in the form of increased numbers of distinct DNA fragments sequenced and can be leveraged in multiple ways. Applying SMURF-seq on a single sample for a full run corresponds to higher counts for downstream analysis. In CNV analysis, increased counts either add confidence for a fixed resolution or can allow higher resolution analysis (i.e., smaller bins) at the same level of confidence. Alternatively, the increased information throughput can effectively reduce the time required to produce the same number of counts for CNV analysis by terminating the sequencing earlier. Finally, the increased information yield can be directed towards reducing the cost of generating CNV profiles by allowing a greater degree of multiplexing. For CNV analysis at resolutions permitted by 250k mapped fragments, our results show SMURF-seq allows roughly 20 and up to 30 samples in a single run, compared with 10 per run directly using short-read sequencing.

CNV analysis using “low-coverage” whole-genome sequencing, at resolution comparable to what we present is becoming increasingly important in diagnostic evaluation of cancer. The loss of tumor-suppressor genes *PTEN* and *RB1* and the amplification of *MYC* oncogene play important roles in prostate cancer prognosis [25]. Bin size determines resolution, and using larger bins reduces capacity for observing smaller events. However, many diagnostic amplifications and deletions of important genes (including loss of *TP53* or amplification of *ERBB2* [26]) are in the megabase size range. For instance, the focal amplification of androgen receptor (AR) [21, 27] and *MYCN* [28] as well as loss of *PTEN* [29] and *RB1* [28] can be detected with CNV profiling using 5000 bins.

Instruments like the MinION are accessible for almost any lab, with a very low buy-in. SMURF-seq allows such technology to be more efficiently leveraged in read-counting applications like CNV analysis. Despite the extreme low buy-in associated with current nanopore-based instruments, the cost per run remains relatively high. This is expected to drop as the technology matures. At the same time, as explained below, improvements to throughput for long-read sequencing on these instruments will directly translate into improved efficiency of SMURF-seq.

The most important factor in the performance of SMURF-seq is that sequencing concatenated fragments effectively eliminates the pore reload time for all but the first fragment in each read. However, there are a variety of additional factors that favor further optimization of the approach employed by SMURF-seq. First, reduction of resources spent on technical nucleotides: SMURF-seq uses a single barcode and sequencing adapter per read consisting of multiple fragments; sequencing short reads uses one barcode and adapter per fragment, adding approximately 50 bases to each fragment. This increases the time to sequence each short read (Additional file 1: Additional text 3.2). In sequencing short reads, as the reads get shorter the time consumed by these technical bases increases. In SMURF-seq, sequencing either shorter fragments in fixed length reads, or longer reads containing fragments of fixed average length, both reduce the time consumed sequencing these technical bases. In the limit, assuming 100-bp DNA fragments, sequencing those fragments as short-reads corresponds to 33% technical nucleotides; for SMURF-seq, the portion of technical nucleotides remains low. Second, more nucleotides sequenced at full speed: We observed that the speed of sequencing was lower when sequencing short molecules. For example, the average sequencing speed was 315.54 bases per second for sequencing the diploid genome without SMURF-seq and 400.29 bases per second when sequencing using SMURF-seq on the MinION sequencer (Additional file 1: Figure S13). Third, leveraging optimizations to long-read protocols: The rapidly evolving nanopore library construction kits are continually optimized for long-read sequencing and would likely require significant ad-hoc modifications to optimize sequencing of short molecules of length optimal for read-counting applications. SMURF-seq alleviates these drawbacks by using the nanopore instrument as intended for long-read sequencing, while generating the desired short fragments.

At present, SMURF-seq has several potential drawbacks. For users already routinely conducting CNV analysis, with established workflows for both wet and dry components, SMURF-seq is likely to present no immediate benefit. Although the restriction enzymes we used do not appear to have introduced substantial bias in our results, using different restriction enzymes could introduce bias and would have to be verified. We have not thoroughly assessed if there might be regions in the genome that are difficult to capture when using SMURF-seq; these need to be assessed when using SMURF-seq for other read-counting applications. Overall driving down the fragment length (to roughly 100 bp) is desirable for SMURF-seq. However, as fragment length decreases, mapping becomes more challenging for both directly sequencing short reads and the SMURF-seq approach, but the impact will be greater for SMURF-seq, due to the intricacy of mapping.

We aligned SMURF-seq reads using the BWA-MEM software [12]. Though not designed for the purpose of aligning SMURF-seq reads, BWA-MEM still accurately identifies the fragments within reads and their genomic mapping locations. At current fragment lengths, for the application of profiling copy number variation (and other read-counting applications), there is little room for improving mapping accuracy. However, with shorter fragments, accuracy in identifying fragment boundaries will begin to impact the ability of aligners to recover fragments, and algorithms designed specifically to map SMURF-seq reads will become essential.

We used SMURF-seq with the low-cost MinION sequencer to obtain data similar to that expected from typical short-read sequencing and generated high-quality CNV profiles from this output. With a fast and simple preparation method and a turnaround time measured in hours, the SMURF-seq approach could provide a highly efficient methodology for research and clinical laboratories where access to large-scale sequencing is limited. We envision a broadening of the applications of SMURF-seq as the underlying sequencing technology evolves and as SMURF-seq itself improves by continual decrease in fragment lengths, increase in sequenced read length, and data analysis methods optimized for SMURF-seq resulting in an increase in information yield per nucleotide sequenced.

## Methods

### DNA samples

The normal diploid female DNA was purchased from Promega (Cat. no. G1521). Breast cancer cell line SK-BR-3 (American Type of Culture Collection (ATCC), Cat. no. HTB-30) was cultured in RPMI-1640 medium (Thermo Fisher Scientific, Cat. no. 11875093) supplemented with 10% fetal bovine serum (FBS) (Thermo Fisher Scientific, Cat. no. 35011CV), was maintained at 37^*circ*^ in a humidified chamber supplied with 5% CO_2_, and was regularly tested for mycoplasma infection.

### Cell lysis and DNA purification

The DNA from SK-BR-3 cells was extracted and purified with the QIAamp DNA Blood Mini Kit (Qiagen, Cat. no. 51104) following the protocol for cultured cells given by the manufacturer. RNA and proteins in the cells were degraded using RNase A stock solution (100 mg/ml) (Qiagen, Cat. no. 19101) and Protease-K (Qiagen, Cat. no. 19133) respectively. Both purchased female diploid DNA and extracted SK-BR-3 DNA were treated with the same downstream processes.

### Fragmenting genomic DNA

Two to 3 *μ*g of genomic DNA was fragmented with restriction enzyme Anza 64 SaqAI (Thermo Fisher Scientific, Cat. no. IVGN0644) for 30 min at 37^*circ*^. The fragmented DNA was cleaned with the QIAquick PCR purification kit (Qiagen, Cat. no. 8106) and eluted with 34 *μ*l nuclease-free water. The concentration of DNA was quantified on a Qubit Fluorometer v3 (Thermo Fisher Scientific, cat. no. Q33216) with the Qubit dsDNA HS assay kit (Thermo Fisher Scientific, cat. no. Q32854).

### Ligation of fragmented DNA

Five hundred nanograms of fragmented DNA in 10 *μ*l nuclease-free water was mixed with 10 *μ*l Anza T4 DNA Ligase Master Mix (Thermo Fisher Scientific, Cat. no. IVGN210-4) and incubated for 30 min at room temperature. The ligated DNA was cleaned with 2 × volume Ampure XP beads (Beckman Coulter, Cat. no. A63881) and eluted in nuclease-free water. This step was done in multiple tubes if more than 500 ng of fragmented DNA was needed to be ligated. The concentration of DNA was quantified on a Qubit Fluorometer v3 with the Qubit dsDNA HS assay kit to ensure ≥ 1 *μ*g (≥ 400 ng, if the Rapid kit was used for library preparation) remained. The size of the ligated DNA molecules were assessed with 1*%* agarose gel electrophoresis run at 90 V for 30 min.

### Library preparation (SQK-LSK108 1D DNA by ligation)

One microgram of re-ligated DNA in 45 *μ*l of nuclease-free water was end-repaired and dA-tailed (New England Biolabs (NEB), Cat. no. E7546), followed by elution in nuclease-free water after 1.5× volume Ampure XP beads clean-up. Sequencing adapters (AMX1D) were ligated with Blunt/TA Ligase Master Mix (NEB, Cat.no. M0367) and cleaned with 0.4× volume Ampure XP beads and eluted using 15 *μ*l Elution Buffer (ELB) following the manufacturer’s protocol (Oxford Nanopore Technologies (ONT), 1D genomic DNA by ligation protocol).

### Multiplexed library preparation (EXP-NBD103 and SQK-LSK108)

Seven hundred nanograms of each re-ligated sample in 45 *μ*l of nuclease-free water was end-repaired, dA-tailed (NEB, Cat. no. E7546), cleaned with 1.5× volume Ampure XP beads, and eluted in nuclease-free water. Different Native Barcodes (NB-x) for each sample was ligated with Blunt/TA Ligase Master Mix (NEB, Cat.no. M0367), cleaned with 2× volume Ampure XP beads and eluted in nuclease-free water. Equimolar amounts of each sample was pooled to have 700 ng of DNA in 50 *μ*l water. Barcode adapters (BAM) were ligated with Quick T4 DNA Ligase (NEB, Cat. no. E6056), cleaned with 0.4× volume Ampure XP beads and eluted using 15 *μ*l Elution Buffer (ELB) following the manufacturer’s protocol (ONT, 1D native barcoding genomic DNA).

### Library preparation (SQK-RAD003 Rapid sequencing)

Four hundred nanograms of re-ligated DNA was concentrated with 2× volume Ampure XP beads to 7.5 *μ*l nuclease-free water. DNA was tagmented with Fragmentation Mix (FRA), and Rapid 1D Adapter (RPD) was attached following the manufacturer’s protocol (ONT, rapid sequencing).

### MinION sequencing and base-calling

All the prepared libraries were loaded on R9.5 Flowcells following the manufacturer’s protocol (ONT) and sequenced for up to 48 h using the script specific to library preparation protocol. Base-calling and de-multiplexing barcoded reads were performed using ONT Guppy (2.3.5) with the appropriate parameters based on the library preparation kit.

### Read alignment

The sequenced reads were mapped to the human reference genome (hg19) using BWA-MEM (0.7.17) with the “-x ont2d -k 12 -W 12 -A 4 -B 10 -O 6 -E 3 -T 120” options (Additional file 1: Additional text 2).

### Estimation of copy number variations

CNV profiles were generated using the procedure described in [19, 20] with the modification employed in [27, 29]. Briefly, the human reference genome (hg19) was split into 5000 (20,000 or 50,000) bins containing an equal number of uniquely mappable locations, and the bin counts were determined using uniquely mapped fragments. Bins with spuriously high counts (“bad bins,” typically around centromeric and telomeric regions) were masked for downstream analysis [20]. This procedure normalizes bin counts for biases correlated with GC content by fitting a LOWESS curve to the GC content by bin count, and subtracting the LOWESS estimate from each bin [20]. Circular binary segmentation (CBS) [30], implemented in DNAcopy [31] package, then identifies breakpoints in the normalized bin counts. Following [27, 29], after CBS, spurious segmentation calls were removed. The influence of the GC content correction can be seen in Additional file 1: Figure S14.

### Comparison with Illumina WGS of SK-BR-3 genome.

DNA from SK-BR-3 cells was used to construct WGS library with the NEBNext UltraII FS DNA Library Prep Kit (NEB, Cat. no. E7805) following the manufacturer’s instructions. After library quality and quantity assessment with Qubit 3.0 HS dsDNA assay and BioAnalyzer HS dsDNA assay (Agilent), libraries were sequenced on HiSeq 2500 (Illumina) with single-end 130 cycles mode.

The reads were mapped with BWA-MEM using the default parameters, PCR duplicates were removed, and CNV profiles were generated using exactly the same method as used for SMURF-seq reads. The scatter plots and Pearson correlations comparing the CNV profiles were produced using R.

## Additional files

Additional file 1 Additional text 1. Supplementary methods. Additional text 2. Mapping SMURF-seq reads. Additional text 3. Short molecule sequencing with long-read sequencers. Additional table 3. Summary of sequencing runs. Figure S1. Distribution of length between restriction sites computed by measuring the distance between the recognition sites on the human reference genome. Figure S2. Schematic of SMURF-seq protocol. Figure S3. Sequencing of restriction enzyme digested normal diploid genome without SMURF-seq. Figure S4. Sequencing normal diploid genome using SMURF-seq. Figure S5. Sequencing normal diploid genome using SMURF-seq with 1D Rapid kit. Figure S6. Sequencing SK-BR-3 cancer genome using SMURF-seq. Figure S7. Replicate sequencing run of normal diploid genome using SMURF-seq. Figure S8. Replicate sequencing run of SK-BR-3 cancer genome using SMURF-seq. Figure S9. High-resolution CNV profile generated using SMURF-seq is highly concordant with the profile generated with Illumina WGS. Figure S10. SMURF-seq generates fragments at a faster rate than sequencing short molecules directly. Figure S11. CNV profile with reads obtained in first few minutes of sequencing. Figure S12. Multiplexed sequencing of normal diploid (barcode01) and SK-BR-3 cancer genome (barcode02) in a single sequencing run. Figure S13. Speed of nanopore sequencing as a function of read length. Figure S14. Biases correlated with GC content are reduced with LOWESS smoothing. (PDF 5703 kb)

Additional file 2 Additional table 1. Alignment score parameter combinations for BWA-MEM, LAST, and Minimap2. Additional table 2. Refining alignment score parameter combinations for BWA-MEM. (XLSX 59 kb)

Additional file 3 Review history. (DOCX 23 kb)

## Data Availability

Scripts and documentation for CNV analysis using SMURF-seq reads and for generating simulated data to evaluate mapping performance are available at https://github.com/smithlabcode/smurfseq_scripts [32] under GNU General Public License version 3 and at Zenodo with the DOI http://dx.doi.org/10.5281/zenodo.3227005 [33]. Sequence data generated during the study are available in SRA with the accession number PRJNA454059 [34]. This work used previously published data (Additional file 1: Additional text 2; Run accession: ERR2184696, ERR2184704, ERR2184712, and ERR2184722) from the study [2] available in the public repository [35].

## Abbreviations

CNV: Copy number variation
SMURF: Sampling molecules using re-ligated fragments
