# Author Correction: SMURF-seq: efficient copy number profiling on long-read sequencers

**DOI:** 10.1186/s13059-020-02149-2

**Published:** 2020-08-25

**Authors:** Rishvanth K. Prabakar, Liya Xu, James Hicks, Andrew D. Smith

**Affiliations:** 1 Quantitative and Computational Biology Section, Department of Biological Sciences, University of Southern California, 1050 Childs Way, Los Angeles, 90089 USA; 2 Michelson Center for Convergent Bioscience, University of Southern California, 1002 Childs Way, Los Angeles, 90089 USA

**Correction to: Genome Biol (2019) 20:134**

**https://doi.org/10.1186/s13059-019-1732-1**

Following publication of the original paper [1], the authors identified an error in the paper.

In the Additional file 1, Figure S4b, both Figure S4a and S4b show the same histogram. This is incorrect. The corrected Additional file 1 is supplied in this correction article.

## Supplementary information

**Additional file 1: Additional text 1.** Supplementary methods. Additional text 2. Mapping SMURF-seq reads. Additional text 3. Short molecule sequencing with long-read sequencers. Additional table 3. Summary of sequencing runs. Figure S1. Distribution of length between restriction sites computed by measuring the distance between the recognition sites on the human reference genome. Figure S2. Schematic of SMURF-seq protocol. Figure S3. Sequencing of restriction enzyme digested normal diploid genome without SMURF-seq. Figure S4. Sequencing normal diploid genome using SMURF-seq. Figure S5. Sequencing normal diploid genome using SMURF-seq with 1D Rapid kit. Figure S6. Sequencing SK-BR-3 cancer genome using SMURF-seq. Figure S7. Replicate sequencing run of normal diploid genome using SMURF-seq. Figure S8. Replicate sequencing run of SK-BR-3 cancer genome using SMURF-seq. Figure S9. High-resolution CNV profile generated using SMURF-seq is highly concordant with the profile generated with Illumina WGS. Figure S10. SMURF-seq generates fragments at a faster rate than sequencing short molecules directly. Figure S11. CNV profile with reads obtained in first few minutes of sequencing. Figure S12. Multiplexed sequencing of normal diploid (barcode01) and SK-BR-3 cancer genome (barcode02) in a single sequencing run. Figure S13. Speed of nanopore sequencing as a function of read length. Figure S14. Biases correlated with GC content are reduced with LOWESS smoothing.
