# Supplementary material for: SMURF-seq: efficient copy number profiling on long-read sequencers
Source: Genome Biol. 2019 Jul 8;20:134. doi: 10.1186/s13059-019-1732-1 (PMC6615205; doi:10.1186/s13059-019-1732-1)
Supplement: Supplementary file 3 — Review history. (DOCX 23 kb) [file 13059_2019_1732_MOESM3_ESM.docx]

**First round of review**

**Reviewer 1**

**Are the methods appropriate to the aims of the study, are they well described, and are necessary controls included?**
Computational mapping strategy deserves attention. See comments below.

**Are the conclusions adequately supported by the data shown?**

The motivation for the concatemerization (rather than just sequencing short reads on the MinION) deserve attention. See comments below.

**Are sufficient details provided to allow replication and comparison with related analyses that may have been performed?**

The code for repeating the analyses should be made available.

**Does the work represent a significant advance over previously published studies? Please explain.**

In spirit, this is significant work for the field of clinical genomics. However, I have some doubts on the advance over just applying MinION seq to the raw (short) reads.

**Is the paper of broad interest to others in the field, or of outstanding interest to a broad audience of biologists?**

No

**Comments to authors**

Prabakar and colleagues present SMURF-seq, a protocol to enable CNV profiling on a MinION sequencer. This is an important subject as this will potentially enable point-of-care, more rapid and cost-effective diagnostic tests. The proposed method is straightforward, the manuscript well-written and to the point. 

Major:
- The important contribution of SMURF-seq is the concatemerization of short fragments into long reads. A fundamental assumption of this work is that this step is better (by some measure e.g. cost, prep time, throughput) than just sequencing short reads on the MinION. I am not sufficiently convinced that is this the case. 
o The authors state (p2, r21) that the protocol involves fewer steps (than what?). Surely it involves more steps than just sequencing short reads on the MinION (even though this may indeed require some (minor) adaptation of the manufacturers protocol, as is argued in Supp text 2.1)? 
o Indeed the MinION will have a bit lower throughput when sequencing short reads and burns a bit more data on "technical" nucleotides (acknowledged by the authors in the Supp text 2.1), but mapping complex long reads that consist of many disjointly mapping fragments to the genome is also reducing useful output. 

The authors state that (abstract): "… which generates multiple copy number profiles in a single run at a reduced time and cost", but don't quantify how much reduction we are talking about. I think this point should be discussed in much more detail in the main text, and the authors should clearly demonstrate (i.e. quantify) that SMURF-seq is indeed significantly better than just putting the short reads on the MinION (again by some measure, not necessarily only throughput). 

- The authors' approach relies on a fragmentation using restriction enzymes with particular recognition sites. This is bound to induce bias, which is not discussed nor normalized for (or shown that this is not necessary). LOESS normalization, as is used by the authors to correct for GC bias, may also be applied (and used to inspect the severity).

- The authors use genome-wide correlation plots to compare their results to the gold-standard (paired-end sequencing). I believe it would be more revealing to use true / false positive rates, and/or report precision / recall of the SMURF-seq profiles compared to the gold standard. Moreover, it is now unclear what the precision of the accuracy of the breakpoints is (which the authors obtain using CBS). Finally, the authors remove "bad bins". It should be specified how this was done, how many were removed, and how this affects the comparison with the ground truth. 

- In line with the previous point, it would be good to have some more close-up figures of chromosomes / chromosome arms, to be able to qualitatively assess the differences between gold standard and SMURF-seq profiles. This may be particularly interesting near regions of the diploid control sample which exhibits 'spikes' (which the authors call a 'flat line'. Moreover, it would be good to analyze if there are regions in the genome that are more difficult to capture using SMURF-seq. 

- The computational approach for mapping the reads is not described in sufficient detail. Figure 1a seems to suggest the restriction site sequence is used in some way (which I believe is a good idea), but the Methods section does not discuss this. Presumably this is because the authors relied on BWA-MEM to split the reads into fragments, which may be problematic especially for shorter fragments. It would be good to consider 'pre-splitting' the reads before mapping (while taking into account that due to sequencing errors restriction sites may be missed and spuriously introduced). 

Minor:
- The availability of the code to analyze SMURF-seq data is not mentioned
- Many figures lack axis labels or specification of the axis units

**Reviewer 2**

**Are the methods appropriate to the aims of the study, are they well described, and are necessary controls included?**
A few controls are missing. A comparison between SMURF-seq and direct sequencing of genomic DNA might be helpful.

**Are the conclusions adequately supported by the data shown?**

Not yet. For example, the quantitative comparisons between SMURF-seq and Illumina CNV profiling should be expanded and the usefulness of sparse WGS in CNV analysis needs to be better justified.

**Are sufficient details provided to allow replication and comparison with related analyses that may have been performed?**

Yes.

**Does the work represent a significant advance over previously published studies? Please explain.**

It could. If the authors do a little more work to justify their conclusions, it could greatly expand the feasibility of WG CNV studies.

**Is the paper of broad interest to others in the field, or of outstanding interest to a broad audience of biologists?**

No

**Comments to authors**

Overall, the SMURF-seq method is an interesting application of long-read sequencing for copy number profiling, and allows for a user to extract more usable data from a MinION device than if they were to use long reads alone. However, the authors should elaborate on the clinical/diagnostic utility of such a method, and provide a more detailed comparison of SMURF-seq to current methods for CNV profiling.

Major:

The authors support the claim that sparse sequencing in CNV detection is accurate by referencing a single-cell sequencing paper. Single-cell methods are greatly constrained by the amount of DNA that is available, which is not the case in this paper. The authors need to support this connection. Could it not be the case that sparse sequencing is not sufficient for a population of cells? For example, we know that the apparent fluctuations in copy number across a population be explained by replication timing [Koren, Cell 2014]. When we look at a single cell this is not as much of a concern. An easy experiment would be to look at deep WGS CNV calls for samples that the author considered and compare them to the sparse method.

The motivation/need for a technique like SMURF-seq should be expanded. The authors discuss particular gene deletions and amplifications as being "prognostic or even predictive for treatment" (page 1, lines 52-3), but the current SMURF-seq protocol uses large bin sizes that are likely only appropriate for identifying large copy number changes and aneuploidies. Do the authors envision SMURF-seq as a tool for identifying these sorts of gross changes/aneuploidies in a clinical context? Or as a tool for rapid CNV profiling of single cells, where starting DNA material is limited? Or something else entirely? This is unclear.

The move away from microarrays and toward WGS for CNV calling was motivated by the need for more precise calls. Since the method proposed here provides lower resolution CNVS (about 600kb), it seems more appropriate to compare the accuracy and availability (e.g., cost, time, etc) of SMURF-seq and microarrays.

Quantitative comparisons between SMURF-seq and Illumina CNV profiling should be expanded. Currently, the authors compare SMURF-seq data to Illumina data by demonstrating concordance between normalized bin counts (Figure 1D, 2E). A more granular comparison might be to directly compare the CNV calls using both methods (i.e., what fraction of duplications/deletions detected by Illumina are recovered by SMURF, and what fraction are private to either method? A simple Venn diagram might suffice). In any case, a more granular comparison would be beneficial.

A comparison between SMURF-seq and direct sequencing of genomic DNA might be helpful. The authors discuss the fact that directly sequencing "short" DNA molecules using Nanopore is inefficient, because short reads contain a higher ratio of "technical" nucleotides and require modification of library protocols to ensure correct stoichiometry (Additional Text 2.1). However, protocols like SQK-RAD003 take very little time, can be used directly on high molecular weight input DNA, and support barcoding. The authors use the SQK-RAD003 kit in conjunction with SMURF-seq, but what do CNV profiles look like if SQK-RAD003 is used directly on genomic DNA without SMURF/re-ligation? The reads output by this protocol will be much longer, and therefore produce fewer individual "mapped fragments" than with SMURF-seq, but does SMURF-seq produce more accurate CNV profiles than SQK-RAD003 alone? Alternatively, how much more efficient is SMURF-seq than SQK-RAD003 alone?

Minor:

The authors claim that the SMURF-seq approach has a "turnaround time measured in hours" (page 3, line 40) though sequencing takes up to 2 days per reaction (page 5, line 12). In addition to sub-sampling sequencing data to 10K reads (Figure 2A), what do the CNV profiles look like if you only use the sequencing data produced during the first 4, 8, 12, etc. hours of the sequencing run? Assuming CNV profiles are concordant between 4 hours and 48 hours' worth of data, this could augment the argument that SMURF-seq is a rapid diagnostic method that requires mere hours to produce results.

How do the predicted distributions of fragment lengths following SaqA1 digestion compare to the mapped fragment lengths following SMURF-seq (i.e., the distributions in Figure S1A and Figure S3B)? It might be interesting to directly compare the expected and recovered lengths of SMURF fragments.

Since the efficiency/accuracy of SMURF-seq depends in part on the sensitivity of the mapper being used, a comparison to another alignment tool would be helpful. What do the CNV profiles look like after aligning the data with minimap2 (https://github.com/lh3/minimap2), for example?

**Reviewer 3**

**Are the methods appropriate to the aims of the study, are they well described, and are necessary controls included?**
The wet lab protocol seems well described. The analysis description is borderline. The analysis code is based on Baslan et al.: Genome-wide copy number analysis of single cells. Nature protocols 7(6), 1024 (2012) and the actual code used to generate the results is not provided. Baslan et al provide their code in the supplementary material of their paper. It uses hardcoded filenames, so substantial work is required if a user actually wanted to use the method. This reduces the accessibility of the method.

**Are the conclusions adequately supported by the data shown?**

Yes.

**Are sufficient details provided to allow replication and comparison with related analyses that may have been performed?**

The absence of actual code used makes this more difficult, but it seems likely.

**Does the work represent a significant advance over previously published studies? Please explain.**

The protocol is neat, but it is not clear to me that the method will be taken up and used in practice.

**Is the paper of broad interest to others in the field, or of outstanding interest to a broad audience of biologists?**

No

**Second round of review**

**Reviewer 1**

The authors have made substantial, albeit mostly textual, improvements to the manuscript. I appreciate their attention to all of the reviewer comments and find most comments have been addressed satisfactorily. Also, the authors now include a more fair discussion of the pros and cons of their method.

An important point which remains an issue for me is the following. The authors point out in their response that they "... made the decision to avoid optimizing computational data analysis procedures in this manuscript." I am surprised by this, as the main (and yes - in my opinion - somewhat limited) contribution of the work presented is a practical implementation of CNV profiling on the MinION. It thus seems counter intuitive that such practical implementation does not come with a 'best practice' computational analysis package. Moreover, the authors concede that they have "several ideas for more sophisticated data analysis". This reviewer is very sensitive to the fact that we cannot wait with publishing until a method is absolutely perfect. At the same time, I think work published in Genome Biology deserves to be based on data analysis methods that have at least some level of sophistication. "Catching the attention of others who might be more capable of optimizing the associated computational methods" is not sufficient in my honest opinion.

**Reviewer 2**

The authors have address all of my concerns.

**Reviewer 3**

The authors have considered the reviewer comments carefully and done a very good job responding to each concern. The manuscript has been substantially improved.

I am satisfied by the responses and changes made to address my comments. The responses to the other reviewers also seem reasonable to me.

The authors have done a good job addressing the advantages and disadvantages of SMURF-seq.

It's great that the authors identified the mismatch between published and available version. Thank you.

I still suspect that the uptake of SMURF-seq will be modest, but may be wrong on this point. However, am persuaded by arguments such as:

"We envision a broadening of the applications of SMURF-seq as the underlying sequencing technology improves and as SMURF-seq itself improves by continual decrease in fragment lengths and increase in fragments per read."

This is an astute perspective and would like to see this added to the conclusion.

While SMURF-seq is comparatively expensive, it is fast, and as argued by the authors, it will be possible for some labs without access to Illumina to set this up easily.

The manuscript is deserving of publication.

**Third round of review**

**Reviewer 1**

I am happy the authors included additional experiments with the mapping strategy. This makes a great addition to the manuscript. I have no further comments.
