# Supplementary material for: Author Correction: SMURF-seq: efficient copy number profiling on long-read sequencers
Source: Genome Biol. 2020 Aug 25;21:214. doi: 10.1186/s13059-020-02149-2 (PMC7447571; doi:10.1186/s13059-020-02149-2)
Supplement: Supplementary file 1 — Additional file 1: Additional text 1. Supplementary methods. Additional text 2. Mapping SMURF-seq reads. Additional text 3. Short molecule sequencing with long-read sequencers. Additional table 3. Summary of sequencing runs. Figure S1. Distribution of length between restriction sites computed by measuring the distance between the recognition sites on the human reference genome. Figure S2. Schematic of SMURF-seq protocol. Figure S3. Sequencing of restriction enzyme digested normal diploid genome without SMURF-seq. Figure S4. Sequencing normal diploid genome using SMURF-seq. Figure S5. Sequencing normal diploid genome using SMURF-seq with 1D Rapid kit. Figure S6. Sequencing SK-BR-3 cancer genome using SMURF-seq. Figure S7. Replicate sequencing run of normal diploid genome using SMURF-seq. Figure S8. Replicate sequencing run of SK-BR-3 cancer genome using SMURF-seq. Figure S9. High-resolution CNV profile generated using SMURF-seq is highly concordant with the profile generated with Illumina WGS. Figure S10. SMURF-seq generates fragments at a faster rate than sequencing short molecules directly. Figure S11. CNV profile with reads obtained in first few minutes of sequencing. Figure S12. Multiplexed sequencing of normal diploid (barcode01) and SK-BR-3 cancer genome (barcode02) in a single sequencing run. Figure S13. Speed of nanopore sequencing as a function of read length. Figure S14. Biases correlated with GC content are reduced with LOWESS smoothing. [file 13059_2020_2149_MOESM1_ESM.pdf]

Supplementary Information
SMURF-seq: efficient copy number profiling on long-read sequencers

Rishvanth K. Prabakar[1], Liya Xu[2], James Hicks[2], and Andrew D. Smith[1*]

[1]Quantitative and Computational Biology Section
[2]Michelson Center for Convergent Bioscience
Division of Biological Sciences, University of Southern California, Los Angeles, California 90089, USA
{*kaliappa,liyaxu,jameshic,andrewds*}@*usc.edu*

Additional text

1 Supplementary methods

1.1 Detailed SMURF-seq protocol

SMURF-seq protocol consists of four steps. Restriction enzyme digestion to fragment DNA, spin-column clean-up to remove restriction enzymes, re-ligation of fragmented DNA with T4 DNA ligase, and Ampure XP beads clean-up to remove ligase enzymes (Fig. S2 and Table 1).

Restriction enzyme digestion
Kit used: Anza 64 SaqAI (Thermo Fisher Scientific, Cat. no. IVGN0644) or Anza 56 Hin1ll (Thermo Fisher Scientific, Cat. no. IVGN0566).

1. Mix the following in a 1.5 ml microcentrifuge tube: Nuclease-free water to make the final volume to 20 µl, 2 µl Anza 10x Buffer, 1 µg sample DNA, and 1 µl Anza restriction enzyme.
2. Mix reagents by pipetting and spin down.
3. Incubate at 37° for 30 min.
4. Repeat the procedure in as many tubes as necessary.

Spin-column clean-up
Kit used: QIAquick PCR purification kit (Qiagen, Cat. no. 28106).

1. Add 125 µl of Buffer PB and mix by pipetting and spin down.
2. Load the sample in a QIAquick column, spin at 13,000 rpm for 1 min and discard the flow-through.
3. Add 750 µl of Buffer PE to the column, spin at 13,000 rpm for 1 min and discard the flow-through.
4. Replace the column in the collection tube and spin again at 13,000 rpm for 1 min to remove any residual wash buffer.
5. Transfer the column to a clean 1.5 ml microcentrifuge tube.
6. Add 34 µl of nuclease-free water to the center of the membrane and wait for 1 min.

Step	Time
RE digestion	∼30 min
Spin-column clean-up	∼15 min
Re-ligation	∼30 min
Ampure XP beads clean-up	∼15 min
Total	∼90 min

Table 1: Steps involved and time needed for SMURF-seq protocol: SMURF-seq protocol involves digesting DNA molecules using restriction enzymes and re-ligating them with DNA Ligase. SMURF-seq protocol takes approximately 90 minutes to complete.

7. Spin the column at 13,000 rpm for 1 min to elute DNA.
8. Quantify the concentration of DNA using a Qubit Fluorometer (Thermo Fisher Scientific, cat. no. Q33216) with the Qubit dsDNA HS assay kit (Thermo Fisher Scientific, cat. no. Q32854).

Ligation of fragmented DNA
Kit used: Anza T4 DNA Ligase Master Mix (Thermo Fisher Scientific, Cat. no. IVGN210-4).

1. Add the following in a 1.5 ml microcentrifuge tube: 500 ng of end-repaired DNA in 10 µl nuclease-free water and 10 µl Anza T4 DNA Ligase Master Mix.
2. Mix the reagents by pipetting and spin down.
3. Incubate at room temperature for 30 min.
4. Repeat the procedure in as many tubes as necessary.

XP beads clean-up
Kit used: AMPure XP beads (Beckman Coulter, Cat. no. A63881).

1. Add 40 µl Ampure XP beads (2× volume) to the ligation reaction and mix by pipetting.
2. Incubate at room-temperature in a hula-mixer for 5 min.
3. Spin down the sample, place the microcentrifuge tube on a magnetic rack and wait for the supernatant to clear. Pipette off the supernatant and discard.
4. Add 200 µl of freshly prepared 70% ethanol and remove the ethanol without disturbing the pellet. Repeat.
5. Remove from magnet, spin down and replace on the magnet. Pipette off any residual supernatant and dry the beads for 1 min.
6. Remove from magnet and re-suspend the beads in 46 µl nuclease-free water. Incubate for 2 min at room temperature.
7. Place on magnet and wait till the elute is clear. Pipette out 46 µl of elute which contains the ligated DNA.
8. Quantify the concentration of DNA using a Qubit Fluorometer with the Qubit dsDNA HS assay kit.

1.2 Fragmentation techniques for SMURF-seq

In the SMURF-seq protocol, we used restriction enzymes to fragment DNA molecules. We also tested acoustic shearing (Covaris, Cat. no. S220) and dsDNA fragmentase (NEB, Cat. no. M0348L) to fragment DNA. These methods increase the time needed for SMURF-seq because of the additional end-repair and wash steps that are required. Moreover, the ligated molecules from these methods were shorter compared with the molecules using the restriction enzyme protocol.

For acoustic shearing, the machine was set to shear DNA to approximately 150 bp (Fig. 1a). After shearing, DNA was end-repaired to have blunt ends and then re-ligated with T4 DNA ligase (Fig. 1b). We

found that repeating the end-repair and re-ligation steps increased the molecule lengths (Fig. 1c). Finally, the ligated molecules were washed with 0.5× volume Ampure XP beads to remove molecules less than 500 bp (Fig. 1d).

For dsDNA fragmentase, the standard protocol provided by the manufacturer was followed to fragment DNA molecules and the reaction was stopped after 30 minutes (Fig. 2a). The molecules were then end-repaired to have blunt ends and re-ligated with T4 DNA ligase (Fig. 2b). Finally, the ligated molecules were washed with 0.5× volume Ampure XP beads to remove molecules less than 500 bp (Fig. 2c).

2 Mapping SMURF-seq reads

2.1 Simulating SMURF-seq reads to evaluate mapping programs

The basic functionality of identifying boundaries between fragments within reads already exists in several mapping tools. To test these, we chose to create simulated reads with the technical characteristics we expect in idealized SMURF-seq data. We first selected a fragment length ℓ and a number k of fragments per read. Then, for a given WGS nanopore data set, we took the set of mapped long reads as determined by BWA-MEM (with `-x ont2d` option). Each of the mapped reads was split into fragments of length ℓ (with a random offset of 0 to $\ell - 1$ at the start of the long read). Each fragment was validated by requiring that it did not overlap a deadzone in the genome (as determined by the deadzone program available from https://github.com/smithlabcode/utils for 40 bp). The reason for excluding deadzones is that even when a short fragment has a "known" mapping location when it is part of a longer read, we cannot compare its reported mapping location as a short fragment with that known location, since we expect any good mapping algorithm to identify that the fragment maps ambiguously. Among these validated fragments, subsets of k were sampled uniformly at random and concatenated (in random order and orientation) to form simulated SMURF-seq reads.

The first and last fragments in a read should be slightly easier to identify and map than the rest, since one of their boundaries is known. Using the above procedure, we select $k = 20$ so that the simulated reads have a sufficient number of fragments to eliminate the influence of the first and last fragments in each read on the results. There is no need to have large k otherwise.

By lowering ℓ and making the fragments shorter, the task of mapping the fragments becomes more challenging. Real SMURF-seq reads have fragment lengths determined by restriction site density, size selection and other aspects of the experiments. But in testing mapping algorithms and optimizing parameters, there is no disadvantage to making the task more challenging. We only need to be able to distinguish the relative performance of different mapping tools and parameter combinations. Real SMURF-seq reads have varying fragment lengths, but in evaluating mapping tools, there is no need to randomize fragment lengths. None of the algorithms we evaluated are capable of either deducing or leveraging the fact that all simulated fragments have the same length. We selected $\ell = 100$, which begins to challenge the various mapping strategies. These values of ℓ are slightly lower than the average in real SMURF-seq data.

2.2 Evaluating performance using simulated SMURF-seq reads

Within the simulated reads, the boundaries of each fragment are known *a priori*, as are their mapping locations. We used this information to evaluate mapping tools in terms of (1) how well they identify fragments purely for the purpose of counting molecules, which is the primary information used in CNV analysis, and (2) how well they identify individual mapping bases within reads. The latter criteria becomes important in challenging cases and will be increasingly important as fragment sizes are reduced.

Performance on identifying fragments: After mapping these simulated reads, each mapping result is called a predicted fragment. Each predicted fragment is considered a positive prediction, and we assume an arbitrary order over positive predictions. A positive prediction is a true positive if:

- The predicted fragment maps uniquely.
- The mapping locations of at least half the bases in the predicted fragment are equal to the original mapping locations for those bases, and those bases are all part of the same original fragment (we assume that it is unlikely for two fragments on a simulated read to have the same mapping location but opposite orientation, and thus do not check for the orientation of a fragment). In this case, we say the predicted fragment is associated with that original fragment.
- The predicted fragment is the first among predicted fragments associated the same original fragment.

False positives are predicted fragments that are not true positives. Any original fragment with no associated predicted fragment is a false negative. These criteria penalize splitting one original fragment or merging two original fragments. By defining true positives, false positives and false negatives we are able to calculate precision, recall, and F-score for a particular mapping strategy.

Performance on identifying individual mapping bases: After mapping simulated reads, each mapping result is decomposed into individual nucleotides and associated with a location in the genome. Those locations are retained. We keep multiplicities, so when two mapped fragments overlap in the genome we count certain nucleotides twice. These are the predicted positive bases in the reference. The condition positive bases are those known a prior from the simulation. The original fragment mapping locations may overlap in the reference genome, leading to multiplicities in the condition positive bases, but with low probability. The true positives are the intersection of the condition positive and the predicted positive bases. When there are multiplicities of mapped fragments and simulated fragments overlapping the same bases in the reference genome, this is determined by taking the smaller of the two values. After removing the true positives bases, the remaining predicted positive bases are false positives, and the remaining condition positive bases are false negatives. These criteria penalize mapping approaches that do not cover the entire simulated SMURF-seq reads, and also penalize approaches that predict fragments that overlap within the read. The true positives, false positives, and false negatives here allow us to assign precision and recall in terms of individual bases and corresponding F-scores. Although the reference bases for both predicted positive and condition positive could involve multisets, since our simulations used relatively low coverage this almost never happened.

2.3 Data sets for generating simulated SMURF-seq reads

To generate simulated reads we used the standard long reads from four sequencing runs (Flowcell ID: FAB42704, FAB42810, FAB49914, and FAF01253) in the public dataset available at https://github.com/nanopore-wgs-consortium/NA12878/blob/master/Genome.md [1, 2]. We downloaded the raw data from EBI (Run accession: ERR2184696, ERR2184704, ERR2184712, and ERR2184722) and base-called these with Guppy (version: 2.3.5).

2.4 Initial selection of mapping tools

We tested the following mapping tools: BWA-MEM[3], Minimap2[4], LAST[5], GraphMap[6], BLASR[7], rHAT[8], and LAMSA[9]. These were selected either because they are known to perform well on certain mapping tasks or have unique properties that plausibly could help in mapping SMURF-seq reads. We tested each of these using default parameters on simulated reads (see above) and downsampled real SMURF-seq reads (data not shown). Among these BWA-MEM, Minimap2, and LAST had higher accuracy on

simulated data, and the other tools identified at most 15 fragments per read on real data. Thus, we explored performance of BWA-MEM (0.7.17), LAST (963), and Minimap2 (2.15) in more detail, varying parameters to improve performance.

We remark that none of these tools were designed to map SMURF-seq reads; results we report here do not reflect the overall performance of the various mapping tools, only that the three aforementioned tools happened to perform relatively well on a task for which they were not directly designed for.

2.5 Detailed evaluation and parameter optimization

The selected mapping tools have variations on the following basic steps:

- Identifying seeds: All tools have a step of identifying seeds, which are short exactly matching parts of the reads. Choices in how seeds are defined and used are often made for mapping speed. The total size of SMURF-seq data sets is currently (relatively) small, so speed is not our primary concern. We favor the most sensitive seed strategy, but depending on implementation too many seed hits could lead to ambiguity later in the mapping process.
- Chaining seeds: The identified seeds are further extended and filtered to avoid aligning potentially false positive seed hits.
- Aligning within the chains: In this stage a Smith-Waterman alignment is performed, typically allowing users to specify a mismatch penalty along with penalties for both gap-open and gap-extend.
- Selecting best alignments: When high-scoring alignments overlap within a read, one of them (or both) could be trimmed or one is selected and the other discarded. The choices made here could lead to discarding entire fragments.

These mapping tools have several parameter options, in general, these are related to: (1) the seeding and chaining algorithm used by the individual tool. (2) The Smith-Waterman alignment scores, i.e. the match score, and the mismatch and indel penalty. The seeding and chaining parameters control the number of proto alignments that are further refined by aligning parts of the read to the reference genome using the specified alignment scores.

The Smith-Waterman alignment score used to align fragments to the reference genome is crucial for determining the optimal fragment length. On one extreme, a match score of 1 with a mismatch and indel penalty of 0 will result in one identified fragment covering the entire read and mapping perfectly, but will always map ambiguously. On the other extreme, a match score of 1 with a mismatch and indel penalty of $-\infty$ will result in any mismatch or indel on the read to be considered as a fragment boundary. Therefore to align SMURF-seq reads, we need to determine optimal alignment scores to use.

In order to determine the optimal alignment score, we kept the seeding related parameters constant, and varied the alignment score combinations to perform a grid search. We varied the mismatch penalty from 1 to 6, gap open penalty from 0 to 4, and gap extend penalty form 1 to 4. The match score was fixed at 1. Thus for each tool we tested 120 ($6 \times 5 \times 4$) combinations of alignment scores.

The seeding and chaining related parameters for each tool was set at follows (along with the four alignment scores):

- BWA-MEM: `-x ont2d -k 12 -W 12 -T 30`
- Minimap2: `-w 1 -m 10 -s 30`
- LAST (NEAR): `lastal -Q0 -e 20` and `last-split -m 1 -s 30`

We set the seeding and chaining parameters in a liberal manner to allow for higher sensitivity than the default parameter of each tool, and the minimum alignment score to output was set at 30.

After aligning the simulated reads, we calculated the average precision and recall, each for the mapped fragment locations and nucleotides, for the four datasets. The F-score was computed for each, and the mean

of the F-scores was used to determine the optimal alignment parameter for each tool. These results for each parameter combinations are reported in Additional file 2: Additional table 1. Based on these results BWA-MEM outperformed other tools for aligning SMURF-seq reads. BWA-MEM performed best with a mismatch, open, and extension penalty of 2, 1, 1 respectively.

To further refine the optimal alignment parameter for BWA-MEM, we aligned the simulated reads with parameter values around the value described above with a higher resolution. We varied the mismatch penalty from 1.5 to 2.5, and open and extend penalties from 0.5 to 1.5 in increments of 0.25. However, BWA-MEM does not accept floating point values for alignment score parameters. To overcome this, we scaled the alignment score proportionately to have integer values, i.e we varied the mismatch penalty from 6 to 10, open and extend penalties from 2 to 6, and fixed the match score at 4 (125 combinations). These results are presented in Additional file 2: Additional table 2. Based on these results, the highest accuracy was obtained with the mismatch, open, and extension penalty of 2.5, 1.5, 0.75 respectively (corresponding scaled values are 10, 6 and 3). We used these optimal alignment scores for mapping real SMURF-seq read, and all the CNV profiles presented are based on these.

3 Short molecule sequencing with long-read sequencers

3.1 Sequencing RE digested normal diploid genome on the Oxford MinION

1 µg of genomic DNA was fragmented with restriction enzyme Anza 64 SaqAI (Thermo Fisher Scientific, Cat. no. IVGN0644) for 30 min at 37°. The fragmented DNA was cleaned with the QIAquick PCR purification kit (Qiagen, Cat. no. 8106) and eluted with 31 µl nuclease-free water. The concentration of DNA was quantified on a Qubit Fluorometer v3 (Thermo Fisher Scientific, cat. no. Q33216) with the Qubit dsDNA HS assay kit (Thermo Fisher Scientific, cat. no. Q32854).

0.5 µg of restriction enzyme digested DNA in 45 µl of nuclease-free water was end-repaired and dA-tailed (New England Biolabs (NEB), Cat. no. E7546), followed by elution in nuclease-free water after 1.5× volume Ampure XP beads clean-up. Sequencing adapters (AMX1D) were ligated with Blunt/TA Ligase Master Mix (NEB, Cat.no. M0367) and cleaned with 1.0× volume Ampure XP beads (manufacturer's protocol uses 0.4× volume XP beads, we increased to 1.0× to get as many short molecules as possible) and eluted using 15 µl Elution Buffer (ELB) following the manufacturer's protocol (Oxford Nanopore Technologies (ONT), 1D genomic DNA by ligation protocol).

The prepared library was loaded on R9.4 Flowcell following the manufacturer's protocol (ONT) and sequenced for 48 hours. Base-calling was performed using ONT Guppy (2.3.5). The sequencing run produced 2.58 million reads with a mean read length of 630.93 bp (Figure S3).

3.2 Limitations of short molecule sequencing with long-read sequencers

There are several disadvantages of sequencing short molecules on a nanopore sequencer:

1. Short molecules have a higher ratio of "technical" to "biological" nucleotides (sequencing adapters and barcodes). In our sequencing run without SMURF-seq, the pores spent 6.1% of their sequencing time on adapters, in comparison to 0.7% when sequencing the diploid genome using SMURF-seq. Defining $d(i)$ as the total duration and $d_t(i)$ as template duration for read i, this is calculated as $1 - (\sum_i d_t(i))/(\sum_i d(i))$ based on information from the sequencing_summary.txt file. This factor will become worse when the samples are barcoded (for example, the barcode kit EXP-NBD103 uses 24 bp barcodes, adding 48 bp to each read), and as the molecules get shorter.

2. The speed of sequencing was lower when sequencing short DNA molecules, likely due to the nanopore requiring a certain number of bases to get to its maximum speed. For example, the average sequencing speed was 315.54 bases per second for sequencing the diploid genome without SMURF-seq, and 400.29 bases per second when sequencing using SMURF-seq (calculated as the mean of $|t(i)|/d(i)$, where $t(i)$ is the template sequence for read i, from the sequencing_summary.txt file; Figure S13). Thus as the molecules get shorter, the nanopores do not sequence at the maximum speed.
3. Our sequencing run produced reads with a mean length of 630.9 bp, which is longer than required for read-counting applications. The continually evolving library preparation protocols would likely require ad-hoc modifications for sequencing molecules of length that are optimal for read-counting applications. Further, certain library preparation kits require long DNA molecules for an optimal library construction, and these kits are not optimal for sequencing short DNA molecules. For example, the rapid sequencing kit uses transposase to fragment DNA molecules and these are not optimal for short DNA molecules as they would fragment the short molecules further.

PacBio machines using the SMRTbell template [10, 11], sequences only one molecule per zero-mode waveguide irrespective of the molecule length. Although, we have not demonstrated this, using SMURF-seq for read-counting applications on a PacBio sequencer will directly lead to an increase in the number of fragments sequenced per read and therefore increase the read-counts obtained from a sequencing run.

Additional table 3: Summary of sequencing runs

Sample	Kit	Reads	Mean length	Fragments	Pores at start
Diploid (SMURF-seq)	SQK-LSK108	270.82k	6.8 kb	7.28M	1453
Diploid (SMURF-seq)	SQK-LSK108	497.92k	3.7 kb	7.55M	1396
SK-BR-3 (SMURF-seq)	SQK-LSK108	146.98k	7.6 kb	4.52M	1175
SK-BR-3 (SMURF-seq)	SQK-LSK108	132.64k	7.3 kb	4.02M	1031
Diploid (SMURF-seq)	SQK-RAD003	213.38k	3.9 kb	2.81M	1461
Multiplexed run (SMURF-seq)	EXP-NBD103 +	442.9k			1060
Diploid (BC01)	SQK-LSK108	138.19k	4.8 kb	2.95M	
SK-BR-3 (BC02)		144.57k	7.7 kb	4.97M	
Diploid (short-read)	SQK-LSK108	2.58M	630.9 bp		1443
SK-BR-3 (WGS)	Illumina WGS	5.56M	130 bp		

SMURF-seq generates substantially more fragments per sequencing run than directly sequencing short reads.

References

[1] Jain, M., Koren, S., Miga, K.H., Quick, J., Rand, A.C., Sasani, T.A., Tyson, J.R., Beggs, A.D., Dilthey, A.T., Fiddes, I.T., et al.: Nanopore sequencing and assembly of a human genome with ultra-long reads. Nature Biotechnology (2018)

[2] Jain, M., Koren, S., Miga, K.H., Quick, J., Rand, A.C., Sasani, T.A., Tyson, J.R., Beggs, A.D., Dilthey, A.T., Fiddes, I.T., et al.: Nanopore sequencing and assembly of a human genome with ultra-long reads. GitHub. Accessed March 25 2019. https://github.com/nanopore-wgs-consortium/NA12878/blob/master/Genome.md

[3] Li, H.: Aligning sequence reads, clone sequences and assembly contigs with bwa-mem. arXiv preprint arXiv:1303.3997 (2013)

[4] Li, H.: Minimap2: pairwise alignment for nucleotide sequences. Bioinformatics **1**, 7 (2018)

[5] Kielbasa, S.M., Wan, R., Sato, K., Horton, P., Frith, M.: Adaptive seeds tame genomic sequence comparison. Genome Research, 113985 (2011)

[6] Sović, I., Šikić, M., Wilm, A., Fenlon, S.N., Chen, S., Nagarajan, N.: Fast and sensitive mapping of nanopore sequencing reads with GraphMap. Nature Communications **7**, 11307 (2016)

[7] Chaisson, M.J., Tesler, G.: Mapping single molecule sequencing reads using basic local alignment with successive refinement (BLASR): application and theory. BMC Bioinformatics **13**(1), 238 (2012)

[8] Liu, B., Guan, D., Teng, M., Wang, Y.: rHAT: fast alignment of noisy long reads with regional hashing. Bioinformatics **32**(11), 1625–1631 (2015)

[9] Liu, B., Gao, Y., Wang, Y.: LAMSA: fast split read alignment with long approximate matches. Bioinformatics **33**(2), 192–201 (2017)

[10] Rhoads, A., Au, K.F.: Pacbio sequencing and its applications. Genomics, Proteomics & Bioinformatics **13**(5), 278–289 (2015)

[11] Goodwin, S., McPherson, J.D., McCombie, W.R.: Coming of age: ten years of next-generation sequencing technologies. Nature Reviews Genetics **17**(6), 333 (2016)

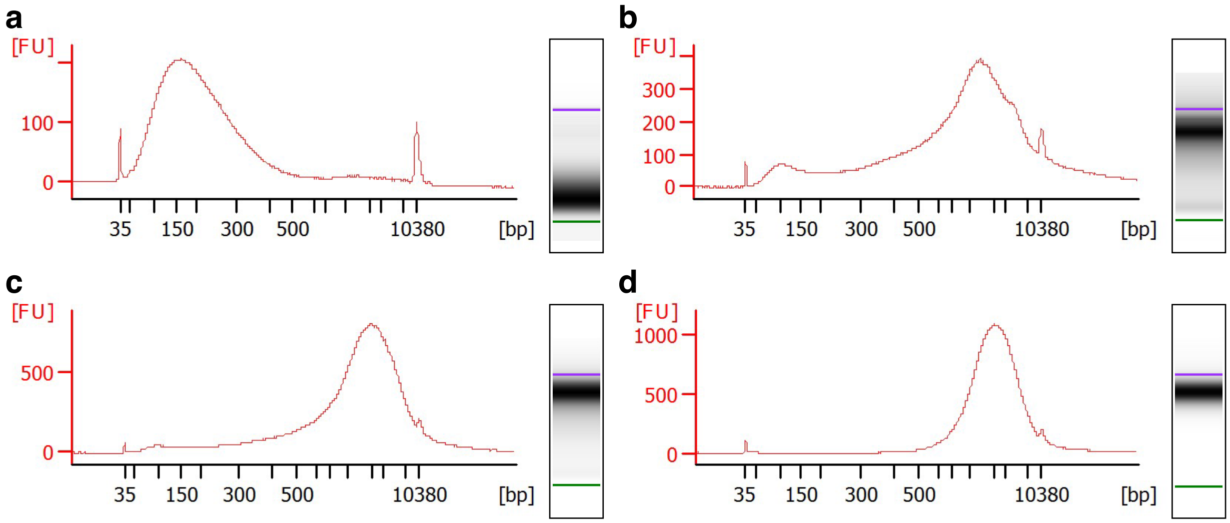

Figure 1: Fragmenting genomic DNA using acoustic shearing. The length distributions were measured using a BioAnalyzer, the "FU" in the plots refer to relative fluorescence units. (a) Length distribution of sheared DNA. (b) Length distribution of re-ligated DNA. (c) Length distribution after second re-ligation. (d) Length distribution of 0.5× volume Ampure XP beads size-selected DNA.

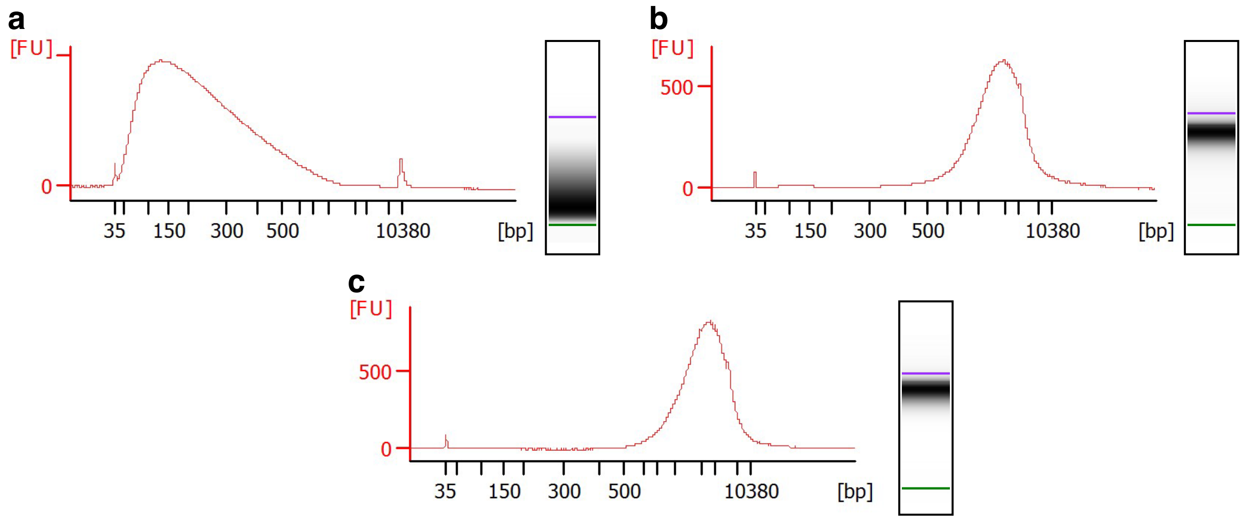

Figure 2: Fragmenting genomic DNA using dsDNA fragmentase enzymes. The length distributions were measured using a BioAnalyzer, the “FU” in the plots refer to relative fluorescence units. (a) Length distribution of fragmented DNA. (b) Length distribution of re-ligated DNA. (c) Length distribution of 0.5× volume Ampure XP beads size-selected DNA.

Supplementary figures

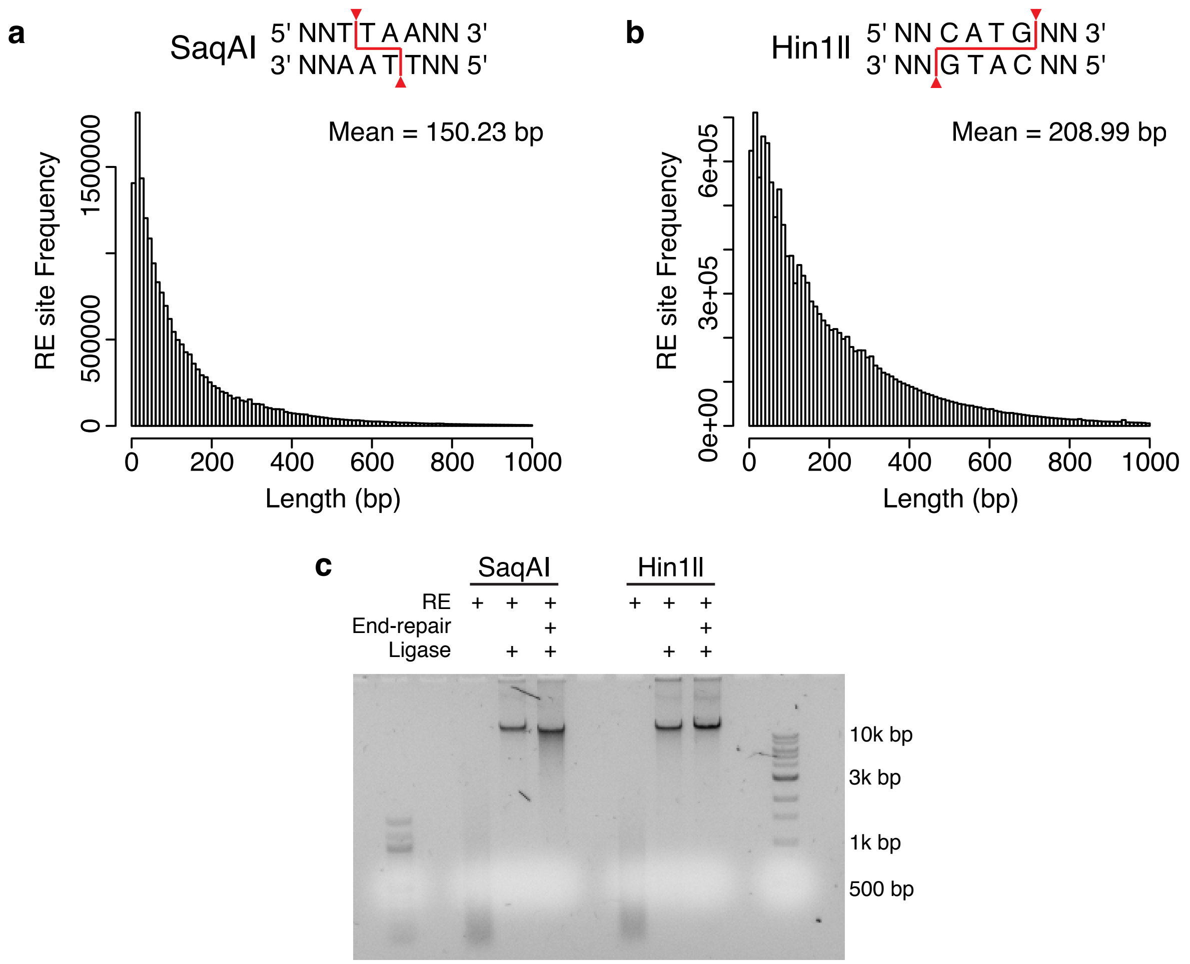

Figure S1: Distribution of length between restriction sites computed by measuring the distance between the recognition sites on the human reference genome. (a) SaqAI recognizes the sequence TTAA and leaves a 2 bp overhang. (b) Hin1ll recognizes the sequence CATG and leaves a 4 bp overhang. (c) Negative gel image of fragmented and ligated normal diploid DNA using restriction enzymes and T4 DNA ligase. Sticky-end and blunt-end ligation (by end-repair) of fragmented DNA are shown, and both yield ligated molecules of approximately the same length.

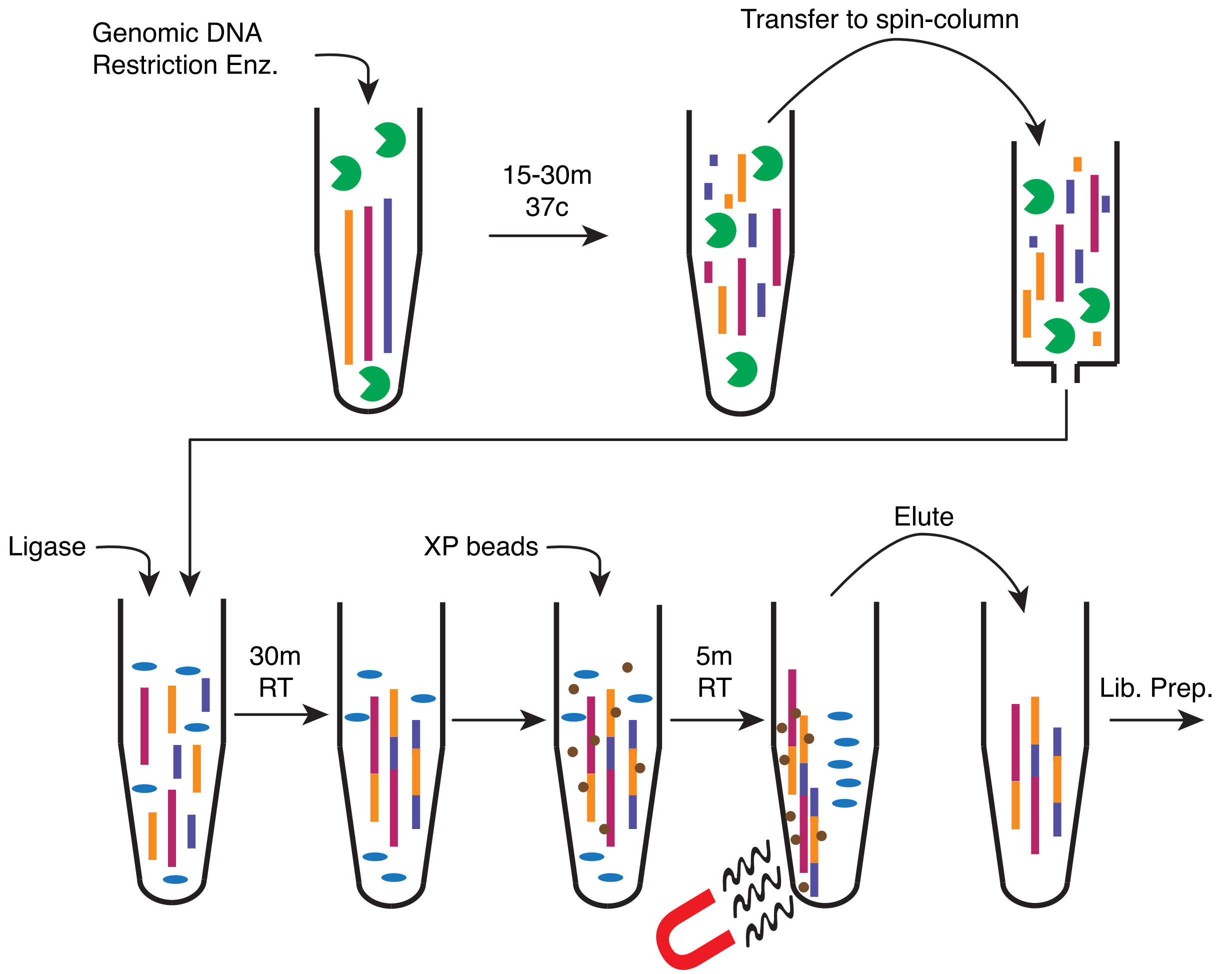

Figure S2: Schematic of SMURF-seq protocol. SMURF-seq consists of four steps: restriction enzyme digestion, spin-column clean-up, re-ligation of fragmented DNA, and Ampure XP beads clean-up.

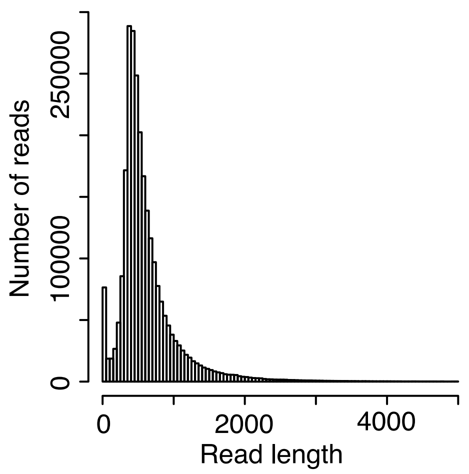

Figure S3: Sequencing of restriction enzyme digested normal diploid genome without SMURF-seq. Sequenced read length distribution (mean read length of 630.93 bp).

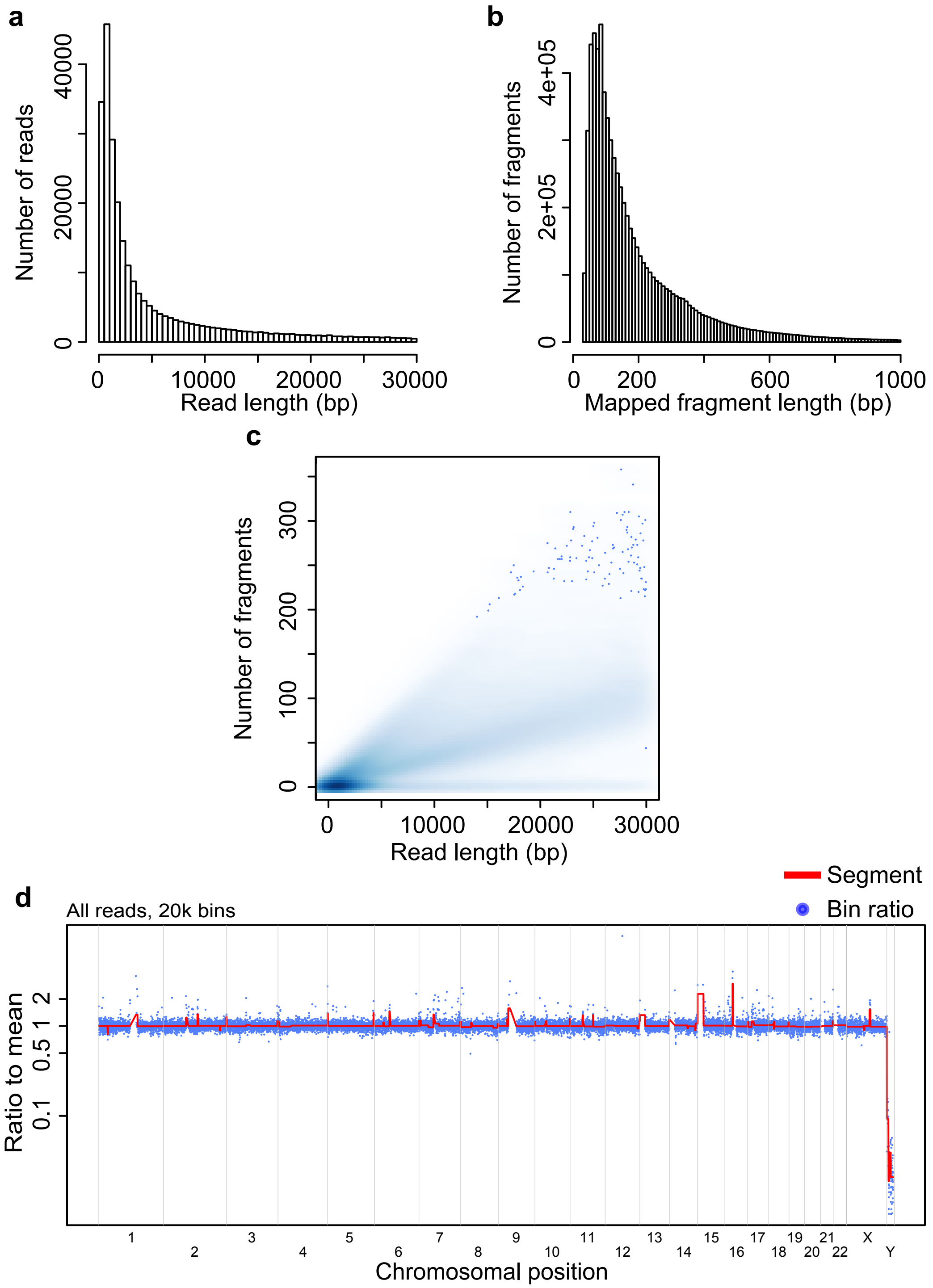

Figure S4: Sequencing normal diploid genome using SMURF-seq. The 270.82k sequenced reads were split into 7.28 million fragments (26.87 mean fragments per read). (a) Sequenced read length distribution (mean read length of 6.8 kb). (b) Mapped fragment length distribution. (c) Scatter plot of read length and the number of fragments contained in the read. (d) CNV profile with 20,000 bins.

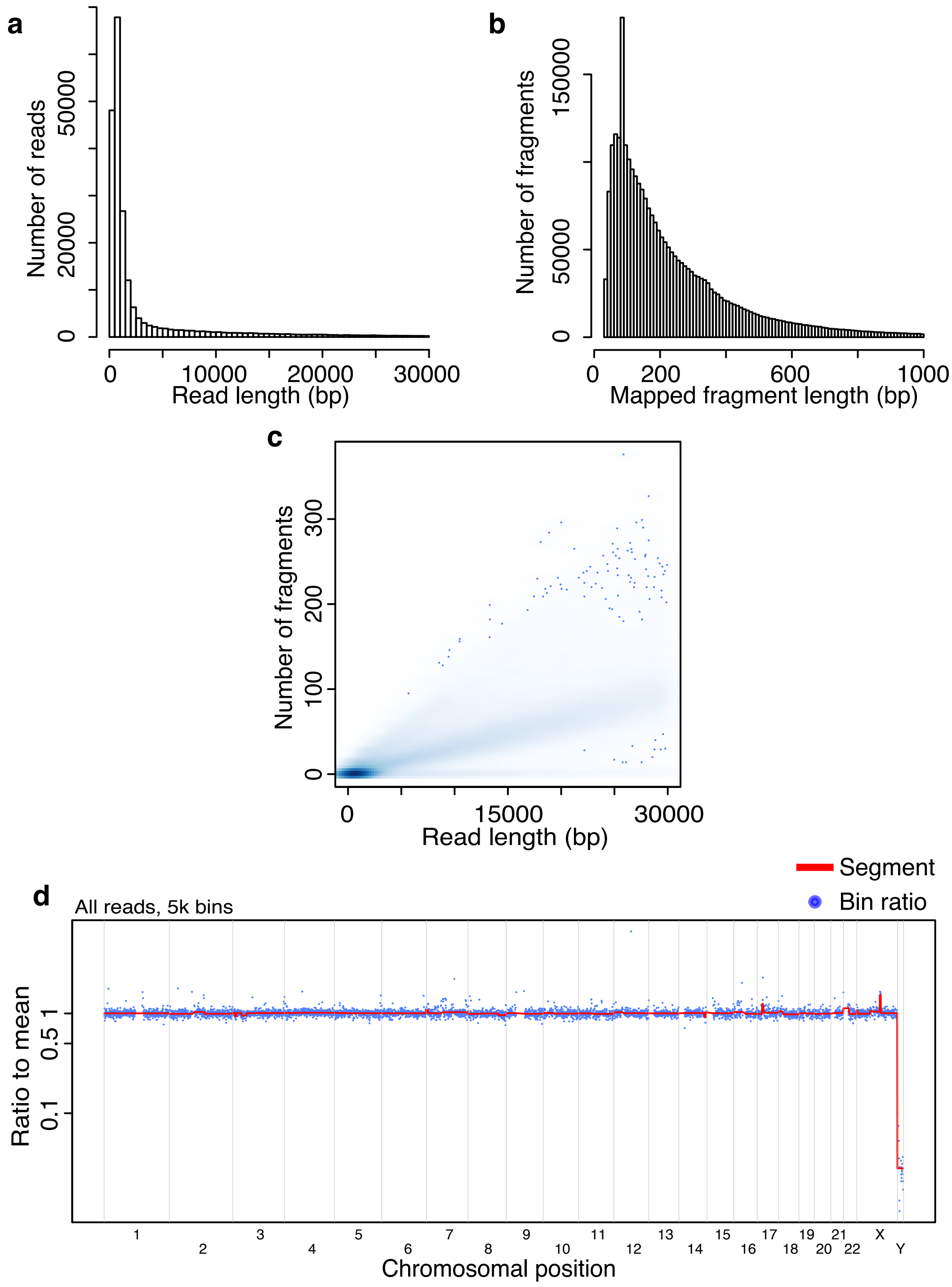

Figure S5: Sequencing normal diploid genome using SMURF-seq with 1D Rapid kit. The 213.38k sequenced reads were split into 2.81 million fragments (13.17 mean fragments per read). (a) Sequenced read length distribution (mean read length of 3.9 kb). (b) Mapped fragment length distribution. (c) Scatter plot of read length and the number of fragments contained in the read. (d) CNV profile with 5,000 bins.

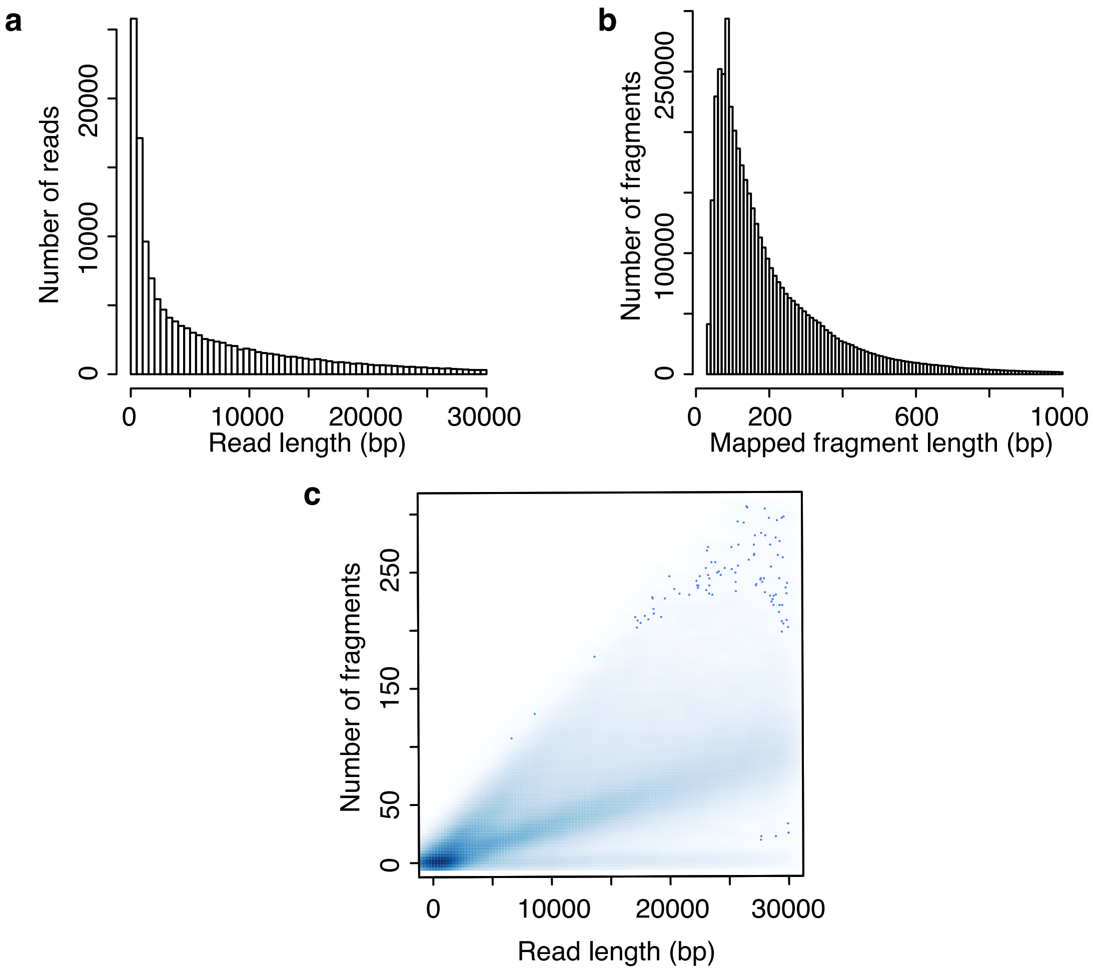

Figure S6: Sequencing SK-BR-3 cancer genome using SMURF-seq. The 146.98k sequenced reads were split into 4.52 million fragments (30.76 mean fragments per read). (a) Sequenced read length distribution (mean read length of 7.62 kb). (b) Mapped fragment length distribution. (c) Scatter plot of read length and the number of fragments contained in the read.

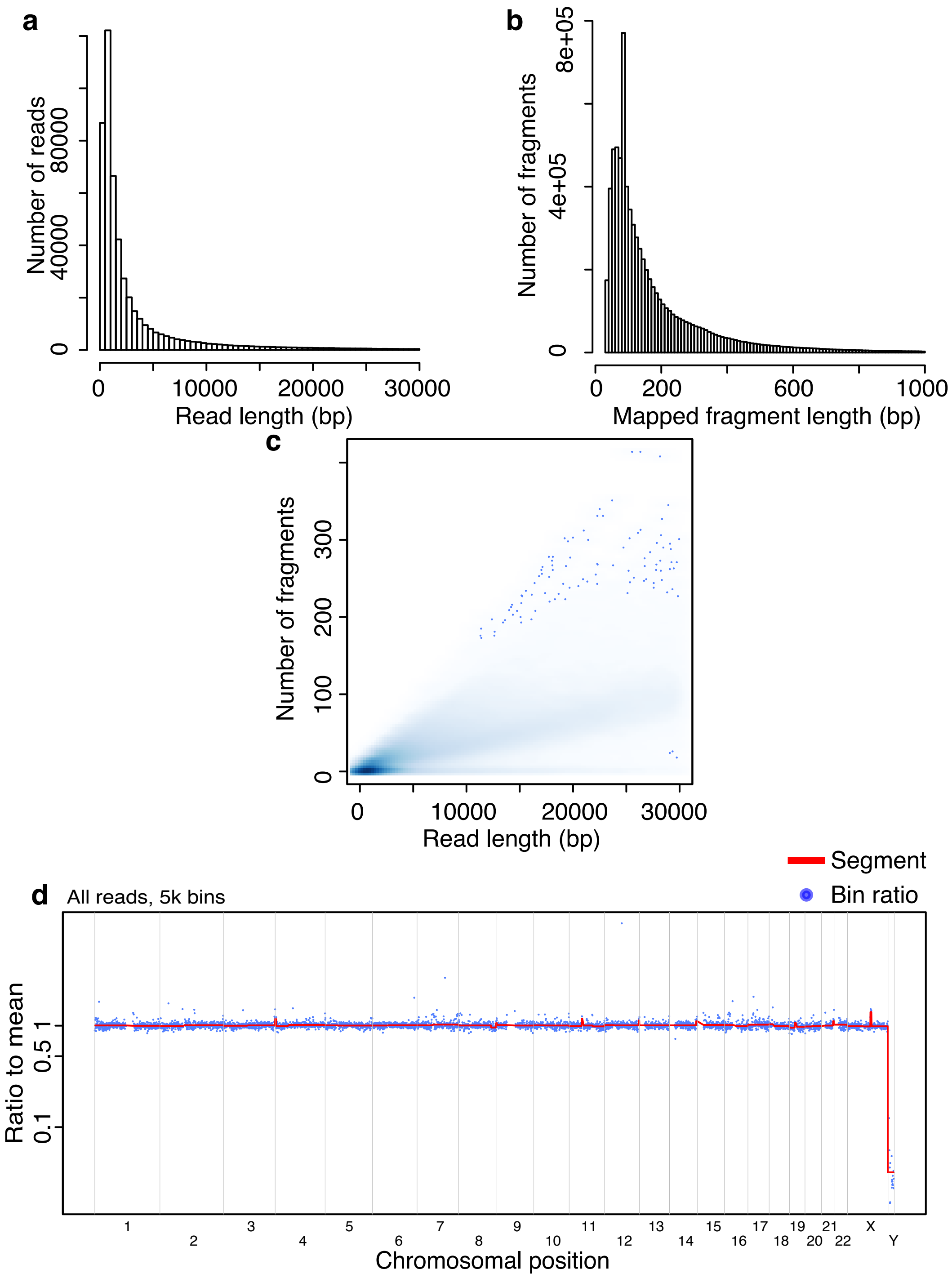

Figure S7: Replicate sequencing run of normal diploid genome using SMURF-seq. The 497.92k sequenced reads were split into 7.55 million fragments (15.17 mean fragments per read). (a) Sequenced read length distribution (mean read length of 3.7 kb). (b) Mapped fragment length distribution. (c) Scatter plot of read length and the number of fragments contained in the read. (d) CNV profile with 5,000 bins.

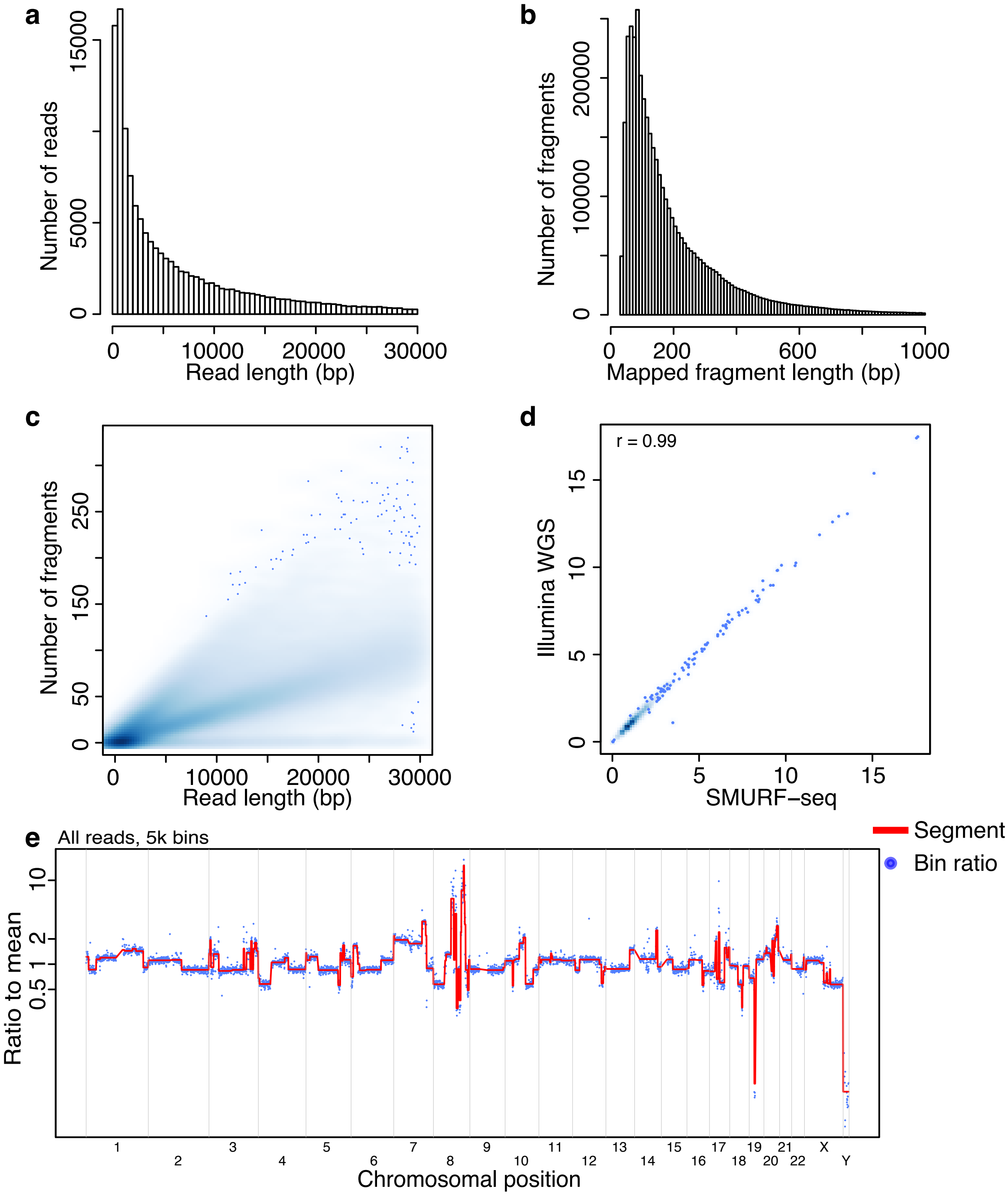

Figure S8: Replicate sequencing run of SK-BR-3 cancer genome using SMURF-seq. The 132.64k sequenced reads were split into 4.02 million fragments (30.32 mean fragments per read). (a) Sequenced read length distribution (mean read length of 7.3 kb). (b) Mapped fragment length distribution. (c) Scatter plot of read length and the number of fragments contained in the read. (d) Scatter plot of bin ratios of SK-BR-3 genome using SMURF-seq and Illumina WGS reads. (e) CNV profile with 5,000 bins.

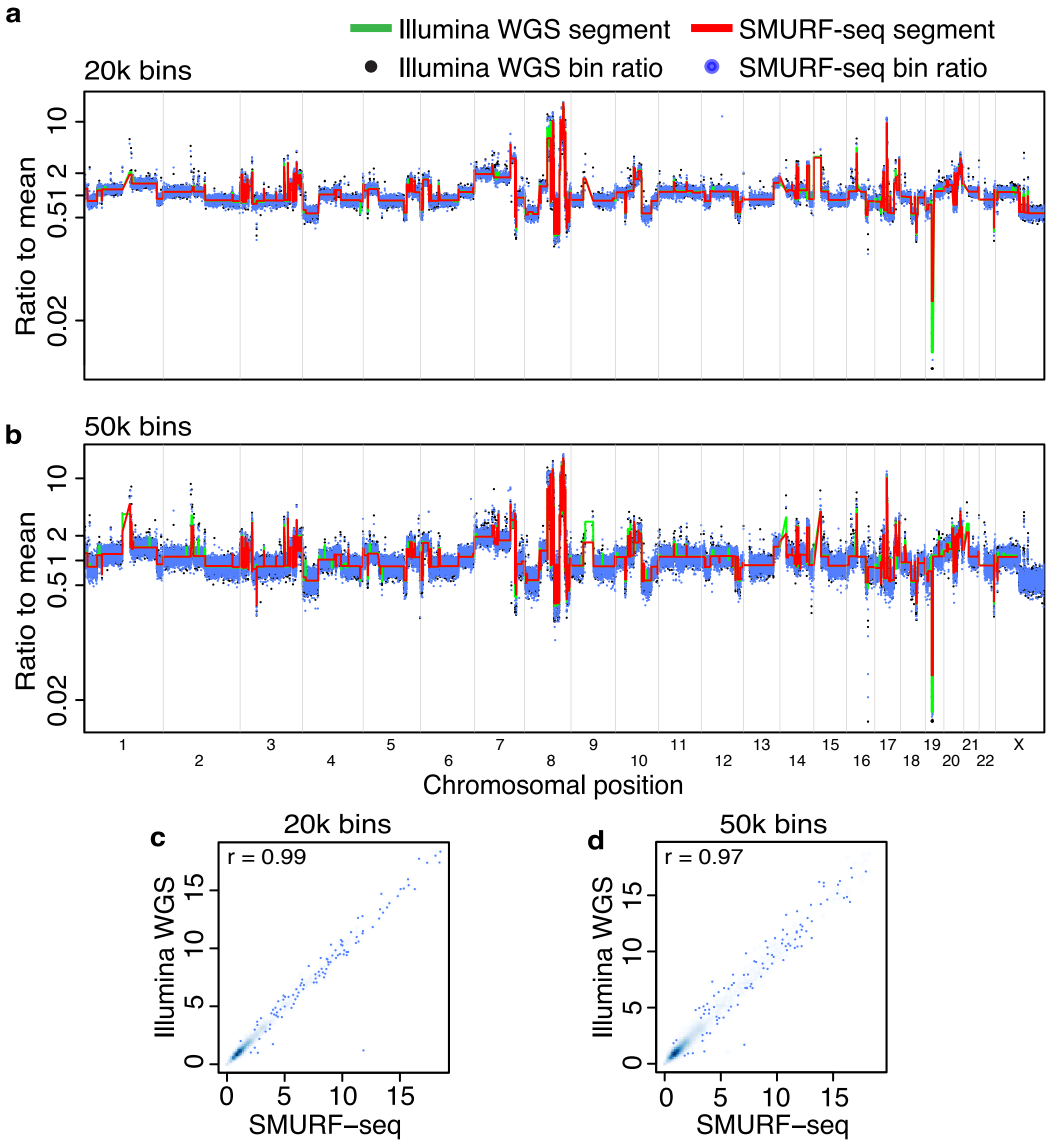

Figure S9: High resolution CNV profile generated using SMURF-seq is highly concordant with the profile generated with Illumina WGS. (a, b) Superimposed CNV profiles of SK-BR-3 genome generated using SMURF-seq and Illumina WGS at 20,000 and 50,000 bin resolutions. (c, d) Scatter plot of bin ratios of SK-BR-3 genome using SMURF-seq and Illumina WGS reads at 20,000 and 50,000 bin resolutions.

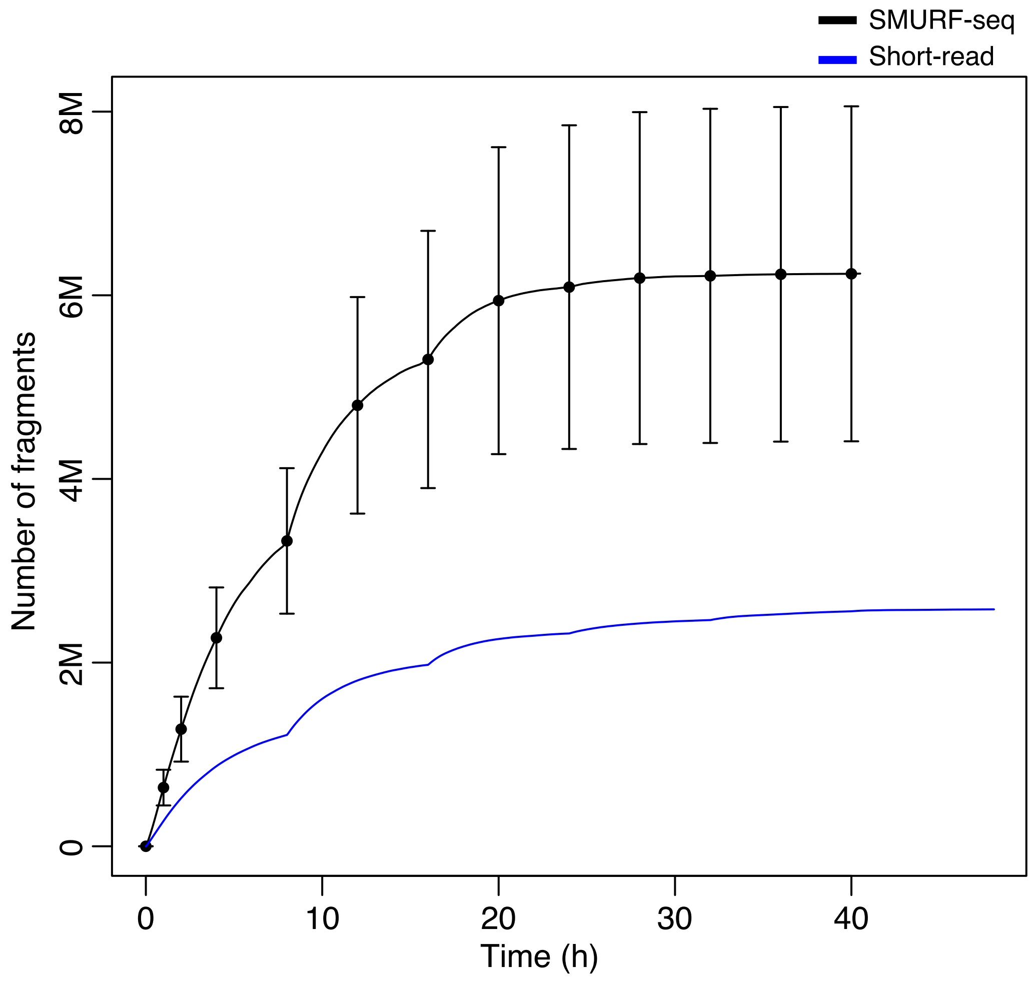

Figure S10: SMURF-seq generates fragments at a faster rate than sequencing short molecules directly. Number of fragments obtained from reads plotted as a function of time. For SMURF-seq, the average number of fragments from runs using the 1D sequencing by ligation kits are plotted (Error bars indicate one standard deviation). For the short-molecule sequencing run, each read is considered as one fragment.

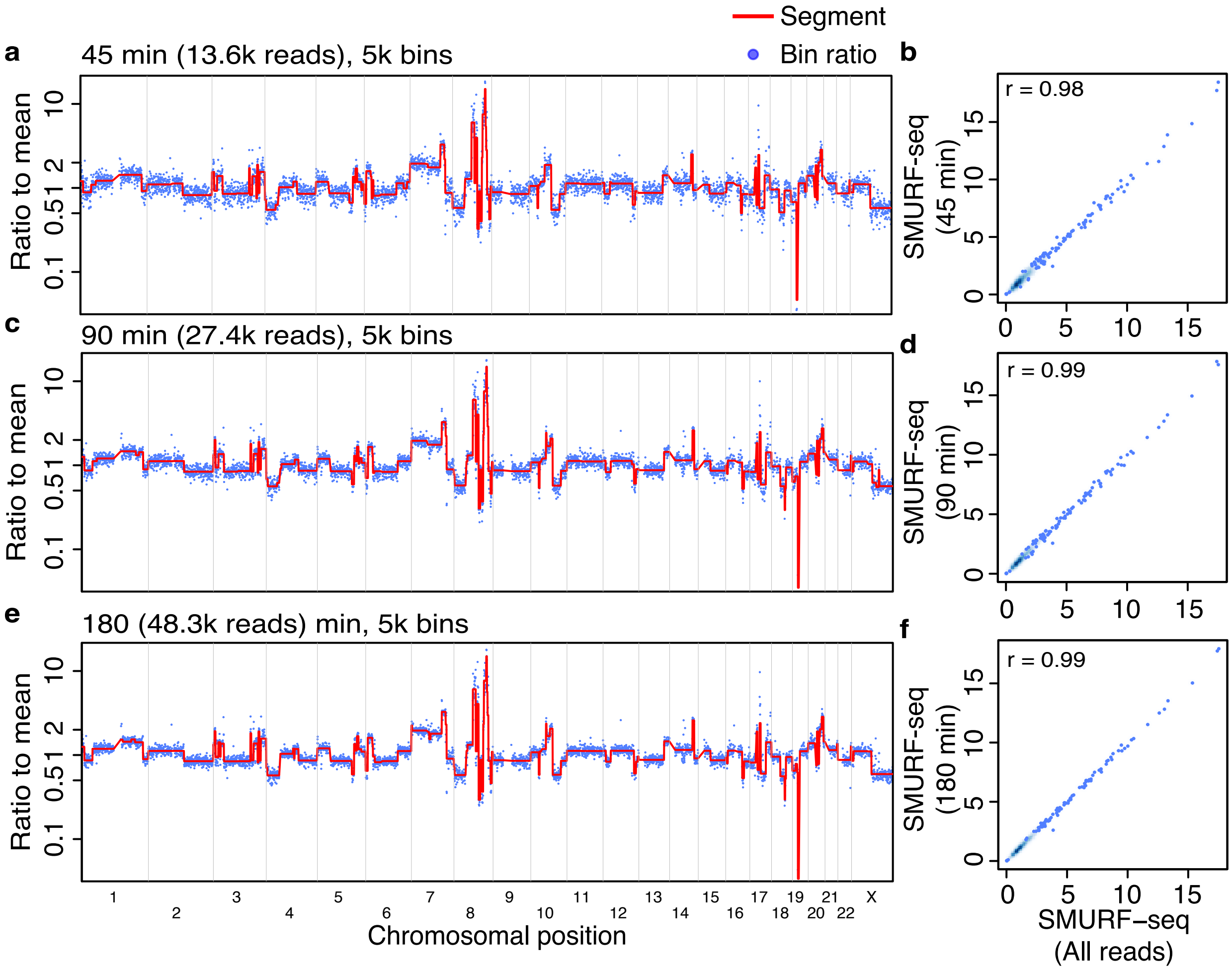

Figure S11: CNV profile with reads obtained in first few minutes of sequencing. (a, c, e) CNV profile with reads obtained in the first 45, 90, and 180 minutes of sequencing. (b, d, f) Scatter plot of bin ratios of the original SMURF-seq data and data obtained in first 45, 90, and 180 minutes of sequencing.

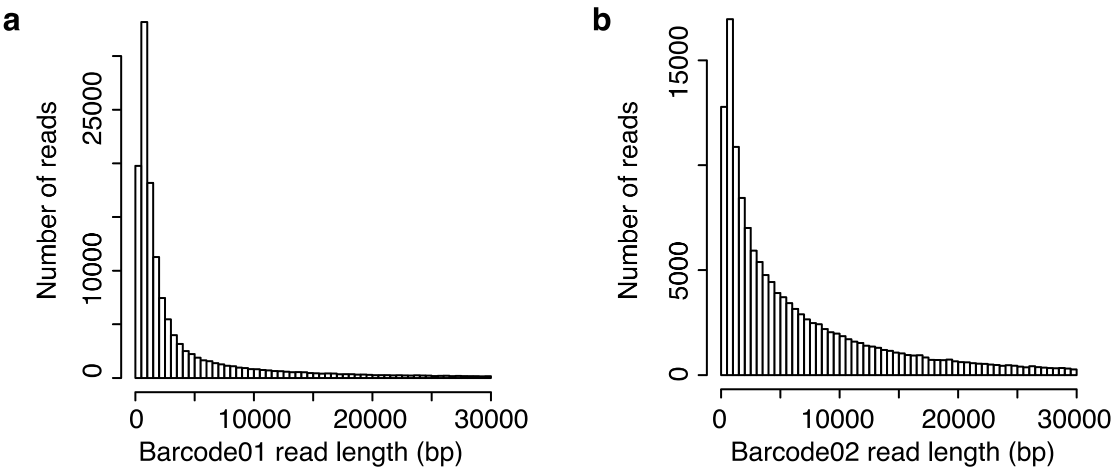

Figure S12: Multiplexed sequencing of normal diploid (barcode01) and SK-BR-3 cancer genome (barcode02) in a single sequencing run. The 138.19k reads with barcode01 were split into 2.95 million fragments (21.34 mean fragments per read) and the 144.57k reads with barcode02 were split into 4.97 million fragments (34.3 mean fragments per read). (a) Sequenced barcode01 read length distribution (mean read length of 4.8 kb). (b) Sequenced barcode02 read length distribution (mean read length of 7.7 kb).

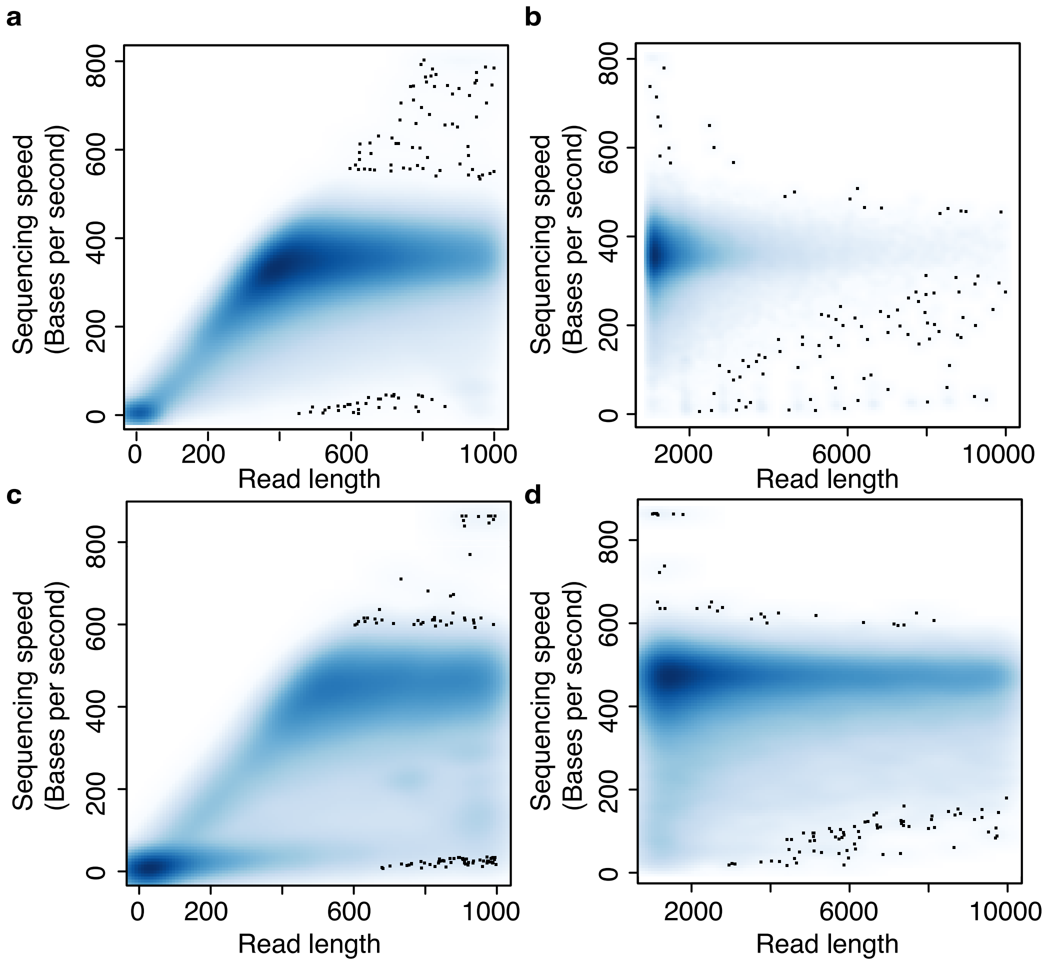

Figure S13: Speed of nanopore sequencing as a function of read length. (a, b) Sequencing speed of diploid genome when sequenced without SMURF-seq. (c, d) Sequencing speed of diploid genome when sequenced with SMURF-seq.

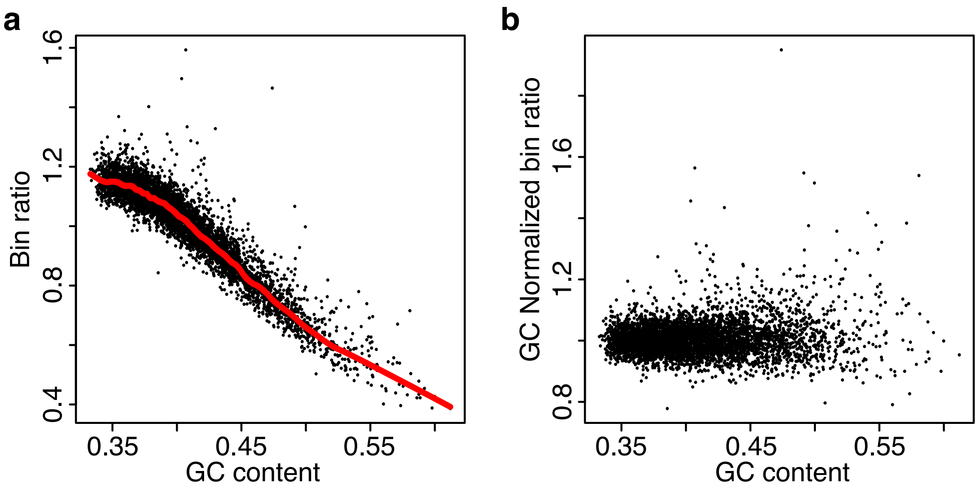

Figure S14: Biases correlated with GC content are reduced with LOWESS smoothing. (a) Bin ratio versus GC content. Red line indicates the LOWESS fit. (b) Bin ratio after bias removal.
